# Supplementary material for: Concurrent and orthogonal gold(I) and ruthenium(II) catalysis inside living cells
Source: Nat Commun. 2018 May 15;9:1913. doi: 10.1038/s41467-018-04314-5 (PMC5954130; doi:10.1038/s41467-018-04314-5)
Supplement: Supplementary file 1 — Supplementary Information [file 41467_2018_4314_MOESM1_ESM.pdf]

SUPPLEMENTARY INFORMATION

Concurrent and orthogonal gold(I) and ruthenium(II) catalysis inside living cells

Cristian Vidal et al

List of Contents

**-Supplementary Methods.**	**3**
**-Supplementary Note 1. Synthesis of gold complexes (Au1 – Au5, Au1' and Au4´).**	**5**
**-Supplementary Note 2. Synthesis of substrates (1,3,5 and 6).**	**9**
**-Supplementary Note 3. Synthesis of products 2 and 4 in organic solvents.**	**12**
**-Supplementary Note 4. Details for the gold-catalyzed hydroarylation in water using procoumarin 1.**	**14**
**-Supplementary Note 5. Gold catalyzed hydroarylation of 3 under aqueous conditions.**	**15**
**-Supplementary Note 6. Characterization of the new compounds.**	**21**
**-Supplementary Note 7. NMR and ESI-MS studies of chloride dissociation in water.**	**28**
**-Supplementary Note 8. Studies of the displacement of $[NTf_2]^-$ in Au4´.**	**36**
**-Supplementary Note 9. Additional NMR studies of the solubility of Au6 complex.**	**37**
**-Supplementary Note 10. General information for the biological experiments.**	**39**
**-Supplementary Note 11. Viability assays.**	**10**
**-Supplementary Note 12. Experiments in living cells.**	**42**
**-Supplementary Note 13. Control reaction between Au1 and substrate 5.**	**46**
**-Supplementary Note 14. ICP analysis.**	**47**
**-Supplementary Note 15. Flow cytometry studies.**	**47**
**-Supplementary Note 16. Estimation of the turnover number in cells.**	**47**
**-Supplementary References.**	**49**

SUPPLEMENTARY METHODS.

Synthetic procedures were performed under an atmosphere of dry nitrogen using vacuum-line and standard Schlenk techniques. Dry solvents were directly purchased from *Sigma Aldrich* and used without further purification. Water used in the catalytic reactions was purchased from *Sigma Aldrich* (LC-MS chromasol) with a pH between 7.4 – 7.6.

Chemicals were purchased from *Sigma Aldrich*, *Alfa Aesar* and *Fluka* and used without further purification.

Gold complexes **Au1-Au5**[1-5] are known compounds and were synthesized from the corresponding ligands following reported procedures. Their ^{1}H and ^{31}P NMR data were in complete agreement with the reported values.

Compounds **1,**[6] **2**[7] and **HXPI** (**6**)[8] are known compounds and were synthesized according to the literature. Their ^{1}H and ^{13}C NMR data were in complete agreement with the reported values.

BSA was purchased from *Sigma Aldrich* as lyophilized powder with purity ≥98% (SKU-A3059). Overnight cultures of bacteria from *E. coli* strain DH5α were grown (OD600=0.3) and were pelleted (3000 xg, 5 min), washed once in PBS and resuspended in 500 µL of PBS.

All catalytic reactions were carried out without particular precautions to extrude moisture or oxygen and open to air except when noted otherwise. Reaction mixtures were stirred using Teflon-coated magnetic stir bars. The abbreviation “r.t.” refers to reactions carried out approximately at 23 °C. Temperature was maintained using Thermowatch-controlled heating blocks. Thin-layer chromatography (TLC) was performed on silica gel plates and components were visualized by observation under UV light. Flash chromatography was carried out on silica gel. Dryings were performed with anhydrous $MgSO_4$.

Concentration refers to the removal of volatile solvents via distillation using a rotary evaporator *Büchi R-210* equipped with a thermostated bath *B-491*, a vacuum regulator *V-850*, followed by residual solvent removal under high vacuum.

^{1}H NMR (300 MHz) spectra were recorded at room temperature on a *Varian Mercury* 300 MHz spectrometer and ^{1}H NMR (500 MHz) spectra were recorded at room temperature on a *Bruker DRX-500* spectrometer. ^{13}C NMR (126 MHz) were recorded on a *Bruker DRX-500* spectrometer and ^{13}C NMR (75 MHz) *Varian Mercury* 300 MHz. ^{31}P NMR (202 MHz) spectra were recorded on a *Bruker DRX-500* spectrometer. ^{19}F NMR (282 MHz) spectra was recorded on a *Varian Mercury* 300 MHz spectrometer. The following abbreviations were used to explain multiplicities: s = singlet, d = doublet, t = triplet, q = quartet, p = pentuplet, h = septuplet, m = multiplet, br = broad signal, bs = broad singlet. NMR spectra were analyzed using *MestreNova© NMR* data processing software (www.mestrelab.com). The chemical shifts (δ) are given in ppm and the coupling constant (J) in Hz.

High resolution mass spectra (HRMS) were acquired using electrospray (ESI) and were recorded at the CACTUS facility of the University of Santiago de Compostela.

Measurements of fluorescence were performed using a *Varian Cary Eclipse* fluorometer. The measurements were made with the following settings: increment 1.0 nm, averaging time 0.1 s, excitation slit width 5.0 nm, emission slit width 5.0 nm, PMT voltage 620 V.

Measurements of UV were performed using a *Jasco V-670* spectrometer.

Electrospray Ionization Mass Spectrometry (ESI/MS) was performed with a *Bruker Amazon IT/MS* using direct injection of a solution of the compound into the MS.

Analytical HPLC was performed, except noted otherwise, on an *Agilent 1260 Infinity II* coupled to an *Agilent Technologies 6120 Quadrupole LC-MS* using a flow rate of 0.35 mL/min at room temperature. For the solvent system was used as initial conditions H_2O/MeCN (50:50) followed by a gradual change over 35 min to H_2O/MeCN (0:100). The chromatogram was recorded via UV absorption at λ = 254 nm.

SUPPLEMENTARY NOTE 1. Synthesis of gold complexes (Au1 – Au5, Au1' and Au4′).

[AuCl(PTA)] (Au1)

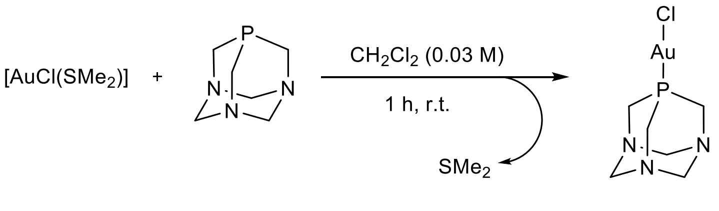

(**Au1**)

Procedure adapted from Besenius *et al.*[1]

[AuCl(SMe$_2$)] (0.339 mmol, 100.0 mg, 1.0 eq.) and CH_2Cl_2 (5.0 mL) were successively added to heat gun dried Schlenk equipped with a stir bar. After the complex was dissolved, PTA (PTA = 1,3,5-triaza-7-phosphaadamantante) was added (0.339 mmol, 0.055 g, 1.0 eq.) followed by CH_2Cl_2 (5.0 mL) and the mixture was stirred at room temperature for 1 h to yield a colorless solution. The solution was then concentrated (to ca. 3.0 mL) in vacuum, and the addition of hexane (ca. 15.0 mL) precipitated a white solid, which was washed with hexane (3 x 15.0 mL) and diethyl ether (3 x 10.0 mL) and dried in vacuum. The gold complex (**Au1**) was isolated as a white solid and stored under nitrogen.

Yield = 85% (0.288 mmol, 112.0 mg).

^{1}H NMR (300 MHz, CD_2Cl_2): δ 4.57 – 4.49 (m, 3H, NCH_2N), 4.42 – 4.32 (m, 9H, NCH_2N / PCH_2N).

^{31}P NMR (202 MHz, CD_2Cl_2): δ -56.5 (s).

[AuCl{κ^1-*S*-(PTA)=NP(=S)(OPh)$_2$}] (Au2)

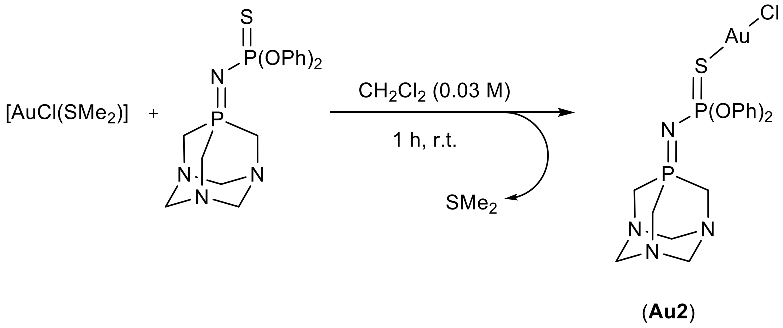

(**Au2**)

Procedure adapted from García-Álvarez *et al.*[2]

[AuCl(SMe$_2$)] (0.270 mmol, 80.0 mg, 1.0 eq.) and CH_2Cl_2 (5.0 mL) were successively added to a heat gun dried Schlenk equipped with a stir bar. After the complex was dissolved,

iminophosphorane ligand was added (0.270 mmol, 114.0 g, 1.0 eq.) followed by CH_2Cl_2 (5.0 mL) and the mixture was stirred at room temperature for 1 h to yield a colorless solution. The solution was then concentrated (to ca. 3.0 mL) in vacuum, and the addition of hexane (ca. 15.0 mL) precipitated a white solid, which was washed with hexane (3 x 15.0 mL) and diethyl ether (3 x 10.0 mL) and dried in vacuum. The gold complex (**Au2**) was isolated as a white solid and stored under nitrogen.

Yield = 91% (0.245 mmol, 160.0 mg).

^{1}H NMR (300 MHz, CD_2Cl_2): δ 7.29 – 7.50 (m, 10H), 4.34 – 4.44 (bs, 12H, 6H for NCH_2N and 6H for PCH_2N).

^{31}P NMR (202 MHz, CD_2Cl_2): δ -31.6 (bs, *P*=N), 38.8 (bs, *P*=S).

[AuCl(PPh$_2$Ph(*p*-CO$_2$H)] (Au3)

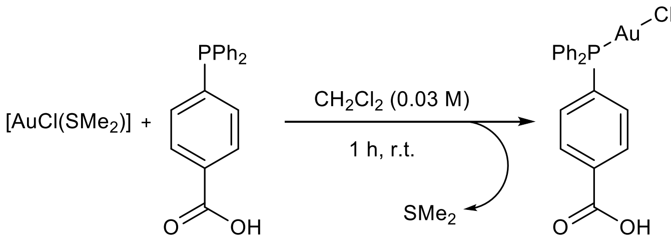

(**Au3**)

Procedure adapted from Monkowius *et al.*[3]

[AuCl(SMe$_2$)] (0.339 mmol, 100.0 mg, 1.0 eq.) and CH_2Cl_2 (5.0 mL) were successively added to a heat gun dried Schlenk equipped with a stir bar. After the complex was dissolved, 4-(diphenylphosphino)benzoic acid was added (0.339 mmol, 100.0 mg, 1.0 eq.) followed by CH_2Cl_2 (5.0 mL) and the mixture was stirred at room temperature for 1 h to yield a colorless solution. The solution was then concentrated (to ca. 3.0 mL) in vacuum, and the addition of hexane (ca. 15.0 mL) precipitated a white solid, which was washed with hexane (3 x 15.0 mL) and diethyl ether (3 x 10.0 mL) and dried in vacuum. The gold complex (**Au3**) was isolated as a white solid and stored under nitrogen.

Yield = 81% (0.274 mmol, 148.0 mg).

^{1}H NMR (300 MHz, CD_2Cl_2): δ 10.75 (br s, 1H), 8.17 – 8.14 (m, 2H), 7.66 – 7.47 (m, 12H).

^{31}P NMR (202 MHz, CD_2Cl_2): δ 34.4 (s).

[AuCl(PPh_2Ph(*m*-SO_3Na))] (Au4)

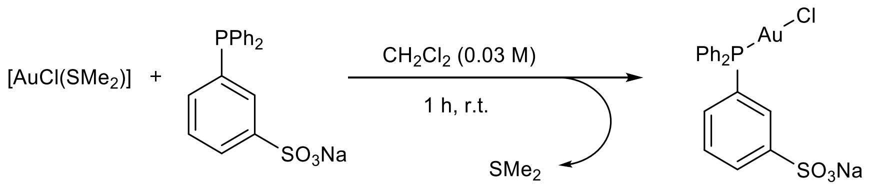

(**Au4**)

Procedure adapted from Laguna *et al.*[4]

[AuCl(SMe_2)] (0.270 mmol, 80.0 mg, 1.0 eq.) and CH_2Cl_2 (5.0 mL) were successively added to a heat gun dried Schlenk equipped with a stir bar. After the complex was dissolved, 3-(diphenylphosphino) benzenesulfonic acid sodium salt was added (0.270 mmol, 98.0 mg, 1.0 eq.) followed by CH_2Cl_2 (5.0 mL) and the mixture was stirred at room temperature for 1 h to yield a colorless solution. The solution was then concentrated (to ca. 3.0 mL) in vacuum, and the addition of hexane (ca. 15.0 mL) precipitated a white solid, which was washed with hexane (3 x 15.0 mL) and diethyl ether (3 x 10.0 mL) and dried in vacuum. The gold complex (**Au4**) was isolated as a white solid and stored under nitrogen.

Yield = 86% (0.232 mmol, 140.0 mg).

^{1}H NMR (300 MHz, CD_2Cl_2): δ 7.79 (d, *J* = 7.5 Hz, 1H), 7.68 (d, *J* = 12.3 Hz, 1H), 6.92 – 7.50 (m, 12H).

^{31}P NMR (202 MHz, CD_2Cl_2): δ 35.3 (s).

[$AuCl_2$(phen)][PF_6] (Au5)

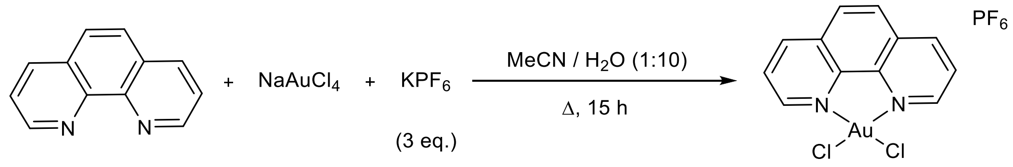

(**Au5**)

Procedure adapted from Ontko *et al.*[5]

$NaAuCl_4$ (0.10 mmol, 40.0 mg, 1.0 eq.), KPF_6 (0.30 mmol, 56.0 mg, 3.0 eq.) and 10.0 mL of H_2O were successively added to a round bottom flask equipped with a stir bar. After the solution was complete homogeneous, a solution of phenantroline (0.10 mmol, 18.0 mg, 1.0 eq.) in MeCN (1.0 mL) was added. The resulting solution was refluxed for 15 h and the colorless hot reaction mixture filtered. The filtrate was washed with H_2O (5 x 5.0 mL) to ensure the complete removal of any unreacted $NaAuCl_4$ and dried in vacuum. The solid was dissolved in acetone (15.0 mL) and heated at 50 °C. This hot solution was then filtered and concentrated (ca. 3.0 mL). Diethyl ether (15.0 mL) was added and a yellow solid precipitated. After removal of the solvent by

decantation, the solid was washed with diethyl ether (3 x 10.0 mL) and dried under vacuum. The gold complex (**Au5**) was isolated as an orange solid and stored under nitrogen.

Yield = 86% (0.086 mmol, 50.0 mg).

^{1}H NMR (300 MHz, DMSO-d_6): δ 9.76 – 9.75 (d, *J* = 6.0 Hz, 2H), 9.41 – 9.39 (dd, *J* = 2.0, 8.0 Hz, 2H), 8.58 (s, 2H), 8.51 – 8.48 (dd, *J* = 6.0, 8.0 Hz, 2H).

[Au(NTf$_2$)(PTA)] (Au1´)

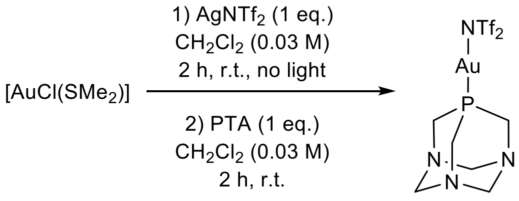

(**Au1´**)

[AuCl(SMe$_2$)] (0.170 mmol, 50.0 mg) and CH_2Cl_2 (5.0 mL) were successively added to a heat gun dried Schlenk equipped with a stir bar. After the complex was dissolved, AgNTf$_2$ was added (0.170 mmol, 65.7 mg, 1.0 eq.). The reaction mixture was stirred at room temperature for 2 h in the absence of light. After that, the solution was filtered over Kieselguhr. To the resulting solution, PTA (0.170 mmol, 26.70 mg, 1.0 eq.) was added and the reaction was stirred at room temperature for 2 h. The solution was then concentrated (to ca. 3.0 mL) in vacuum, and the addition of hexane (ca. 15.0 mL) precipitated a white solid, which was washed with hexane (3 x 15.0 mL) and diethyl ether (3 x 10.0 mL) and dried in vacuum. The gold complex (**Au1´**) was isolated as a white solid and stored under nitrogen.

Yield = 77% (0.131 mmol, 83.10 mg).

^{1}H NMR (300 MHz, DMSO-d$_6$): δ 4.59 – 4.49 (m, 3H, NC*H*$_2$N), 4.43 – 4.33 (m, 9H, NC*H*$_2$N / PC*H*$_2$N).

^{13}C NMR (125 MHz, DMSO-d$_6$): δ 72.5 (*C*H$_2$), 51.3 (*C*H$_2$).

^{31}P NMR (202 MHz, DMSO-d$_6$): δ -44.2 (s).

^{19}F NMR (282 MHz, DMSO-d$_6$): δ -77.92.

ESI-MS (MALDI, DCTB matrix): Calculated for $C_8H_{12}AuF_6N_4O_4PS_2Na$: 656.94995, found 656.95192 $[M+Na]^+$ and 604.19185 [M-NTf$_2$+DCTB].

[Au(NTf$_2$)(PPh$_2$Ph(*m*-SO$_3$Na)] (Au4´)

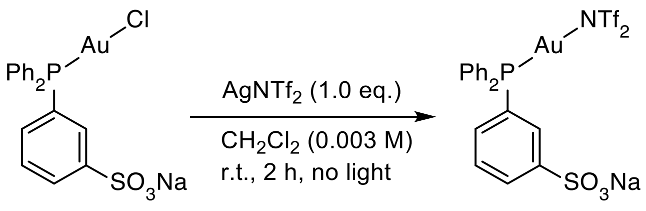

(**Au4´**)

Procedure adapted from Gagosz *et al.*[6]

[AuCl(PPh$_2$Ph(*m*-SO$_3$Na))] (0.050 mmol, 30.0 mg) and CH_2Cl_2 (15.0 mL) were successively added to a heat gun dried Schlenk equipped with a stir bar. After the complex was dissolved, AgNTf$_2$ was added (0.050 mmol, 19.4 mg, 1.0 eq.) and the mixture was stirred at room temperature in the absence of light for 2 h. After that, the solution was filtered over Kieselguhr to remove AgCl. The solution was then concentrated (to ca. 3.0 mL) in vacuum, and the addition of hexane (ca. 15.0 mL) precipitated a white solid, which was washed with hexane (3 x 15.0 mL) and dried in vacuum. The gold complex (**Au4´**) was isolated as a white solid and stored under nitrogen.

Yield = 83% (0.041 mmol, 34.92 mg).

^{1}H NMR (300 MHz, MeOD-d$_4$): δ 8.22 – 8.18 (m, 1H), 8.10 – 8.06 (m, 1H), 7.70 – 7.59 (m, 9H).

^{13}C NMR (125 MHz, MeOD-d$_4$): δ 148.7 (t, *J* = 4.8 Hz, *C* ipso), 137.1 (t, *J* = 7.1 Hz, *C*H), 136.0 (t, *J* = 7.6 Hz, *C*H), 134.3 (s, *C*H), 133.2 (t, *J* = 8.7 Hz, *C*H), 131.9 – 130.93 (m, *C*H), 130.3 (t, *J* = 28.9 Hz), 129.0 (t, *J* = 29.8 Hz, *C* ipso), 121.6 (q, *J* = 320.3 Hz, *C* ipso, *C*F$_3$).

^{31}P NMR (202 MHz, MeOD-d$_4$): δ 46.4 (s).

^{19}F NMR (282 MHz, MeOD-d$_4$): δ -77.3.

SUPPLEMENTARY NOTE 2. Synthesis of substrates (1, 3, 5 and 6).

Synthesis of 3-(diethylamino)phenyl 3-phenylpropiolate (1)

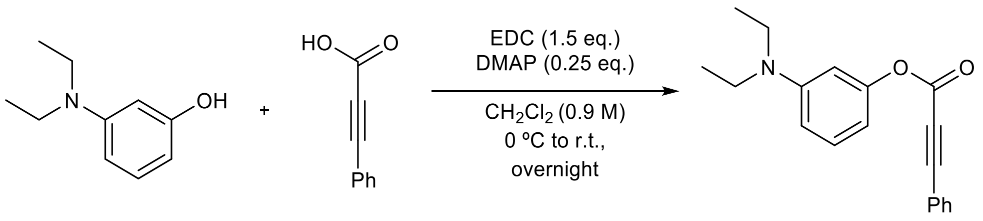

(1.2 eq.) (**1**)

Procedure adapted from Kim *et al.*[7]

3-Dimethylaminophenol (6.00 mmol, 0.991 g, 1.20 eq.), 3-phenylpropiolic acid (5.00 mmol, 0.730 mg, 1.0 eq.), EDC (7.50 mmol, 1.437 g, 1.50 eq.) and DMAP (4-(dimethylamino)pyridine, 1.25 mmol, 152.70 mg, 0.25 eq.) were dissolved in CH_2Cl_2 (5.556 mL, 0.90 M) at 0 °C. The clear black solution was stirred during 1 h at this temperature and then at r.t. overnight. The organic phase was washed with 0.5 N of HCl, $NaHCO_3$ sat. and brine. The organic phase was dried and concentrated in vacuum. The residue was purified by flash column chromatography using hexane / EtOAc (8:2) as eluent. The product **1** was obtained as a light yellowish oil.

R_f = 0.57 (Hexane / EtOAc 8:2).

Yield = 50% (3.0 mmol, 0.733 g).

^{1}H NMR (300 MHz, $CDCl_3$): δ 7.65 (d, *J* = 6.8 Hz, 2H), 7.50 (t, *J* = 7.3 Hz, 1H), 7.44 (dd, *J* = 7.4 Hz, *J* = 1.2 Hz, 2H), 7.25 (t, *J* = 8.1 Hz, 1H), 6.59 (dd, *J* = 6.0 Hz, 1H), 6.48 (dd, *J* = 8.4 Hz, 2H), 3.40 (q, *J* = 7.0 Hz, 4H), 1.20 (t, *J* = 7.0 Hz, 6H).

^{13}C NMR (75 MHz, $CDCl_3$): δ 152.69, 151.59, 149.12, 133.26, 131.01, 130.06, 128.75, 119.58, 109.75, 107.90, 104.50, 88.44, 80.76, 44.78, 12.90.

Synthesis of 2,3,6,7-tetrahydro-1H,5H-pyrido[3,2,1-ij]quinolin-8-yl 3-phenylpropiolate (3)

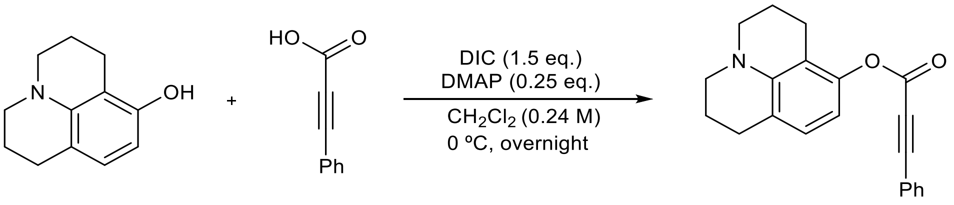

(1.3 eq.) (**3**)

Procedure adapted from Sames *et al.*[8]

Phenyl propiolic acid (5.495 mmol, 0.803 g, 1.3 eq.) and CH_2Cl_2 (6.4 mL) were successively added to a heat gun dried round bottom flask equipped with a stir bar under nitrogen. The mixture was

stirred at 0 °C in an ice/brine bath for 1 min. Then, neat DIC (*N*,*N*-dicyclohexylcarbodiimide, 6.341 mmol, 0.993 mL, 1.5 eq.) was added via syringe and the mixture was stirred for 1 min until a white precipitate was formed. A solution of 8-hydroxyjulolidine (4.227 mmol, 0.80 g, 1.0 eq.) in CH_2Cl_2 (10.0 mL) was added via syringe followed by DMAP (1.057 mmol, 0.129 mg, 0.25 eq.) in CH_2Cl_2 (1.0 mL). The mixture was stirred at 0 °C until complete consumption of the phenol was observed by TLC. Then the reaction mixture was filtered through Kieselguhr and concentrated in vacuum. The residue was purified by flash column chromatography using hexane / EtOAc (8:2) as eluent to yield the product **3** as a yellow/orange solid.

R$_f$ = 0.78 (Hexane / EtOAc 7:3).

Yield = 67% (2.83 mmol, 0.89 g).

^{1}H NMR (500 MHz, $CDCl_3$): δ 7.63 (d, *J* = 8.3 Hz, 2H), 7.48 (t, *J* = 7.5 Hz, 1H), 7.40 (t, *J* = 7.7 Hz, 2H), 6.81 (d, *J* = 8.1 Hz, 1H), 6.34 (d, *J* = 7.9 Hz, 1H), 3.14 (q, *J* = 5.7 Hz, 5H), 2.75 (t, *J* = 6.5 Hz, 2H), 2.65 (t, *J* = 6.6 Hz, 2H), 1.97 (h, *J* = 6.2 Hz, 5H).

^{13}C NMR (126 MHz, $CDCl_3$): δ 152.65, 146.86, 144.10, 133.28, 130.97, 128.73, 127.07, 119.76, 119.68, 119.60, 113.51, 108.51, 88.08, 80.58, 50.01, 49.54, 27.64, 22.01, 21.73, 21.33.

LRMS (*m/z*, ESI): 340.13 $[M+Na]^+$, 318.14 $[M+H]^+$, 129.03.

HRMS-ESI Calculated for $C_{21}H_{19}NO_2$: 318.1489, found 318.1487.

Synthesis of (E)-2-(2-(6-hydroxy-2,3-dihydro-1H-xanthen-4-yl)vinyl)-3,3-dimethyl-1-propyl-3H-indol-1-ium iodide (6, HXPI)

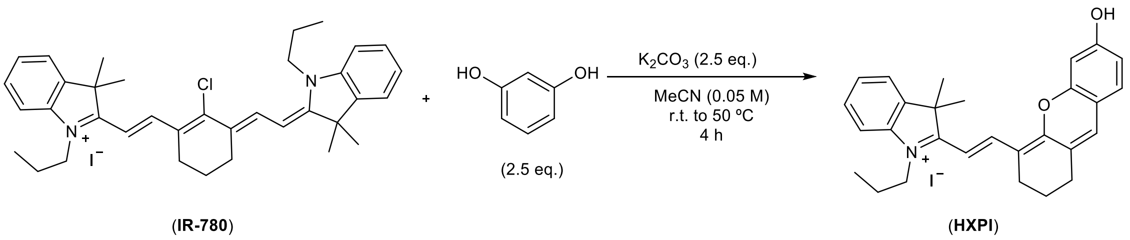

Procedure adapted from Ma *et al.*[9]

Resorcinol (1.870 mmol, 206.0 mg, 2.50 eq.) and K_2CO_3 (1.870 mmol, 259.0 mg, 2.50 eq.) were mixed in a heat gun dried bottom flask containing MeCN (12.0 mL), and the mixture was stirred at r.t under nitrogen atmosphere for 10 min. Then, a solution of IR-780 iodide (0.749 mmol, 500.0 mg) in MeCN (3.0 mL) was added to the mixture via syringe, and the reaction mixture was heated at 50 °C for 4 h. Then, the solvent was evaporated under reduced pressure, and the residue was purified by silica gel chromatography using hexane / CH_2Cl_2 / MeOH (25:25:5) as eluent, affording the HXPI **(6)** as a blue solid.

R$_f$ = 0.13 (Hexane / CH_2Cl_2 / MeOH 25:25:5).

Yield = 20% (0.149 mmol, 60.5 mg).

^{1}H NMR (500 MHz, $CDCl_3$): δ 8.03 (d, *J* = 14.2 Hz, 1H), 7.28 − 7.23 (m, 3H), 7.17(d, *J* = 9.2 Hz, 1H), 7.04 (t, *J* = 7.6 Hz, 1H), 6.81 (d, *J* = 7.6 Hz, 1H), 6.74 (d, *J* = 8.8 Hz, 1H), 6.51 (s, 1H), 5.61 (d, *J* =

14.2 Hz, 1H), 3.76 (t, *J* = 7.2 Hz, 2H), 2.67 (t, *J* = 6.0 Hz, 2H), 2.61 (t, *J* = 6.0 Hz, 2H), 1.91 − 1.78 (m, 4H), 1.66 (s, 6H), 1.06 (t, *J* = 7.6 Hz, 3H).

^{13}C NMR (126 MHz, $CDCl_3$): δ 176.4, 162.9, 154.5, 145.1, 141.8, 141.7, 136.3, 129.0, 128.7, 126.6, 125.7, 122.8, 116.3, 114.9, 114.6, 111.8, 103.2, 102.1, 77.5, 77.2, 76.8, 50.5, 47.0, 29.0, 28.7, 24.4, 21.2, 20.5, 11.8.

Synthesis of (E)-2-(2-(6-(allyloxy)-2,3-dihydro-1H-xanthen-4-yl)vinyl)-3,3-dimethyl-1-propyl-3H-indol-1-ium iodide (5)

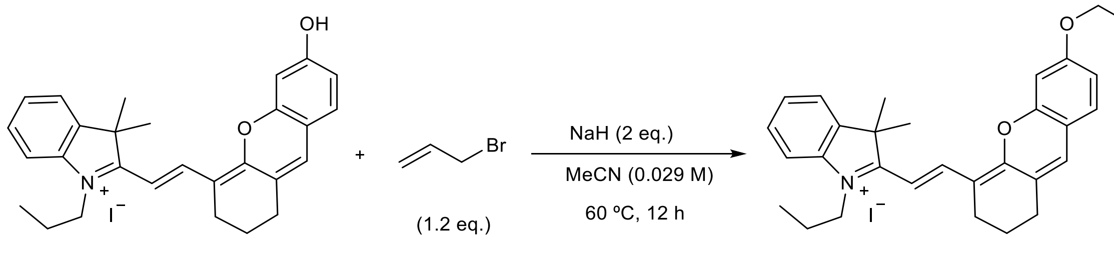

Procedure adapted from Ma *et al.*[9]

To a solution of HXPI (0.09 mmol, 50.0 mg) in dry MeCN (3.20 mL) in a heat gun dried round bottom flask equipped with a stir bar was added NaH (0.185 mmol, 7.40 mg, 2.0 eq) and the solution was stirred at room temperature for 10 min. Then, allyl bromide was added (0.110 mmol, 0.010 mL, 1.2 eq.) via syringe and the resulting mixture was stirred at 60 °C for 12 h under nitrogen atmosphere. After that, the solvent was removed under reduced pressure. The residue was purified by flash column chromatography using CH_2Cl_2 / MeOH (9:1) as eluent. The product **5** was obtained as a blue-green solid.

R_f = 0.07 (CH_2Cl_2 / MeOH 9:1).

Yield = 22% (0.02 mmol, 11.9 mg).

^{1}H NMR (300 MHz, $CDCl_3$): 8.78 (d, *J* = 14.7 Hz, 1H), 7.67 (d, *J* = 7.5 Hz, 1H), 7.58 (m, 2H), 7.50 (m, 2H), 7.44 (m, 1H), 7.09 (m, 1H), 7.02 (dd, *J* = 8.5 Hz, 2.4 Hz, 1H), 6.53 (d, *J* = 13.4 Hz, 1H), 6.14 (m, 1H), 5.51 (dd, *J* = 17.3, 1.6 Hz, 1H), 5.36 (dd, *J* = 10.6, 1.3 Hz, 1H), 4.75 (d, *J* = 4.1 Hz, 2H), 4.36 (t, *J* = 8.2 Hz, 2H), 2.78 (m, 4H), 1.97 (m, 4H), 1.29 (m, 5H), 1.11 (m, 4H).

LRMS (*m/z,* ESI): 452.3 $[M-I]^+$.

HRMS-ESI Calculated for $C_{31}H_{34}NO_2$: 452.2590, found 452.2583.

SUPPLEMENTARY NOTE 3. Synthesis of products 2 and 4 in organic solvent.

Synthesis of 7-(diethylamino)-4-phenyl-2H-chromen-2-one (2a)

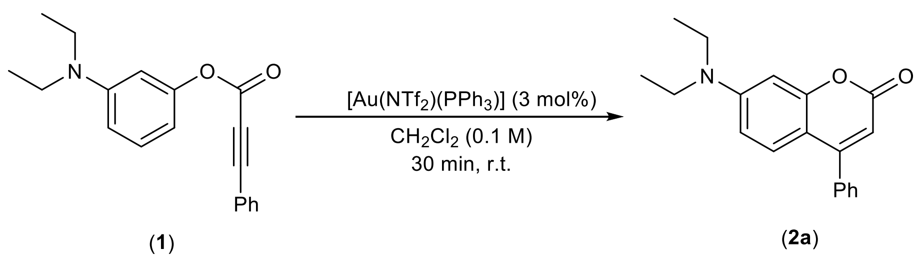

Procedure adapted from Sames *et al.*[8]

Substrate **1** (0.095 mmol, 27.0 mg) was dissolved in dry CH_2Cl_2 (1.0 mL) followed by addition of [bis(trifluoromethanesulfonyl)imidate](triphenylphosphine)gold(I) (2:1) toluene adduct[vi] (0.03 mmol, 4.0 mg, 3.0 mol%). The reaction was stirred at room temperature under nitrogen until complete consumption of the starting material. Then the crude was filtered over Kieselguhr and the purity was evaluated by ^{1}H-NMR. If necessary, the crude was purified by flash column chromatography using hexane / EtOAc (8:2) as eluent. The product (**2a**) was obtained as an orange oil.

R_f = 0.23 (Hexane / EtOAc 5:1).

Yield = 99% (0.94 mmol, 26.0 mg).

^{1}H NMR (300 MHz, $CDCl_3$): δ 7.46 - 7.44 (m, 5H), 7.24 (d, *J* = 8.9 Hz, 1H), 6.54 (d, *J* = 2.4 Hz, 1H), 6.51 (dd, *J* = 8.9 , 2.4 Hz, 1H), 5.97 (s, 1H), 3.39 (q, *J* = 6.9 Hz, 4H), 1.20 (t, *J* = 6.9 Hz, 6H).

^{13}C NMR (75 MHz, $CDCl_3$): δ 162.12, 156.79, 156.16, 150.63, 136.19, 129.25, 128.62, 128.32, 127.92, 108.56, 108.15, 107.83, 97.75, 44.74, 12.43.

Synthesis of 9-phenyl-2,3,6,7-tetrahydro-1H,5H,11H-pyrano[2,3-f]pyrido[3,2,1-ij]quinolin-11-one (4)

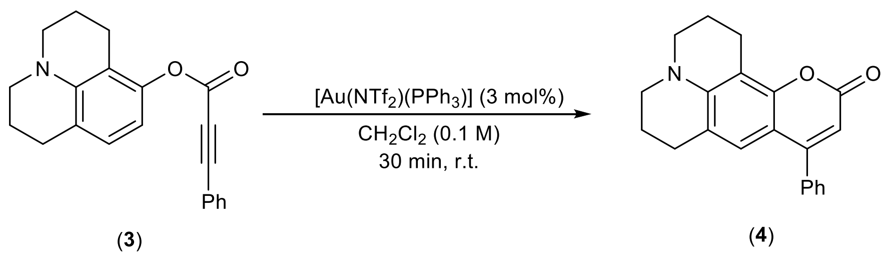

Procedure adapted from Sames *et al.*[8]

Substrate **3** (0.095 mmol, 30.0 mg) was dissolved in dry CH_2Cl_2 (1.0 mL) followed by addition of [bis(trifluoromethanesulfonyl)imidate](triphenylphosphine)gold(I) (2:1) toluene adduct[vi] (0.030 mmol, 4.0 mg, 3 mol%). The reaction was stirred at room temperature under nitrogen until complete consumption of the starting material. Then, the crude was filtered over Kieselguhr and the purity was evaluated by ^{1}H-NMR. If necessary, the crude was purified by flash column chromatography using hexane / EtOAc (8:2) as eluent to yield the product **4** as a yellow solid.

R_f = 0.38 (Hexane / EtOAc 7:3).

Yield = 92% (0.087 mmol, 27.0 mg).

^{1}H NMR (500 MHz, $CDCl_3$): δ 7.51 – 7.44 (m, 3H), 7.43 – 7.38 (m, 2H), 6.81 (s, 1H), 5.96 (s, 1H), 3.26 (t, *J* = 4.9 Hz, 3H), 2.94 (t, *J* = 6.5 Hz, 2H), 2.66 (t, *J* = 6.4 Hz, 2H), 1.99 (p, *J* = 6.3 Hz, 2H), 1.93 (p, *J* = 6.1 Hz, 2H).

^{13}C NMR (126 MHz, $CDCl_3$): δ 162.53, 156.48, 151.89, 146.01, 136.83, 129.10, 128.67, 128.54, 124.09, 118.20, 107.95, 107.13, 50.09, 49.68, 27.78, 21.67, 20.83, 20.69.

LRMS (*m/z, ESI*): 340.13 $[M+Na]^+$, 318.14 $[M+H]^+$.

HRMS-ESI Calculated for $C_{21}H_{19}NO_2$: 318.1489, found 318.1490.

SUPPLEMENTARY NOTE 4. Details for the gold-catalyzed hydroarylation in water using procoumarin 1.

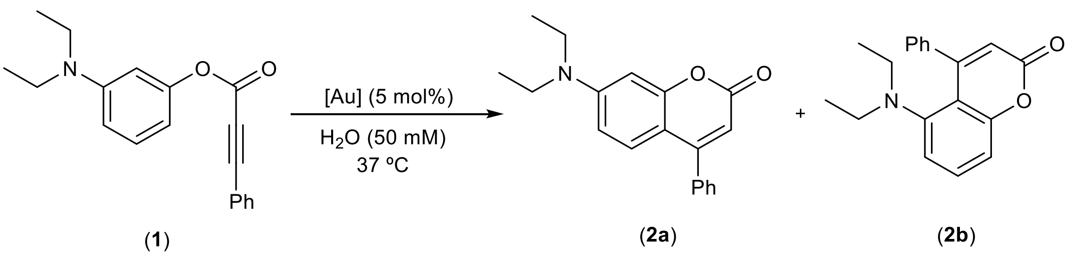

Substrate **1** (0.050 mmol, 14.69 mg) was added to a Schlenk tube containing a stir bar followed by the addition of the corresponding gold complex (0.0025 mmol, 5 mol%). H_2O (1.0 mL) was added and the Thermowatch-controlled heating block was fixed at 37 °C and the reaction was stirred for 24 h. The reaction mixture was extracted with CH_2Cl_2 (3 x 10.0 mL) and the combined organic fractions were dried, concentrated and the crude was analyzed by ^{1}H-NMR using CH_2Br_2 as internal standard.

Supplementary Table 1. Hydroarylation of **1** in water using different gold complexes

Entry	[**Au**]	Yield[a]	Ratio **2a/2b**[a]
1	**Au1**	70	1.5/1
2	**Au2**	70	1.5/1
3	**Au3**	90	10/1
4	**Au4**	90	2.1/1
5	**Au6**	0	-
6	**Au7**	99	1.7/1

[a] Yield and ratio of the regioisomeric coumarins obtained in the hydroarylation reaction using different gold complexes in water.

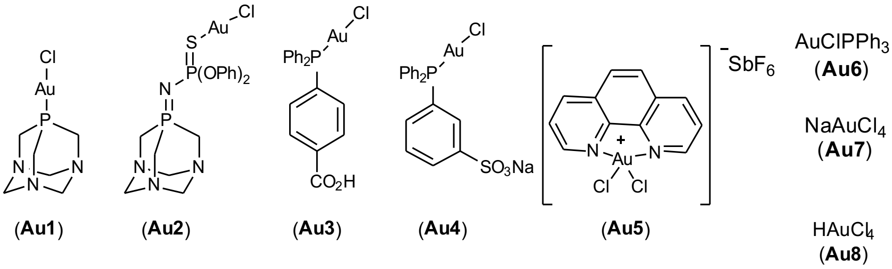

SUPPLEMENTARY NOTE 5. Gold catalyzed hydroarylation of 3 under aqueous conditions.

Representative general procedure for the catalytic hydroarylation of 3 in water using different gold complexes (exemplified for the use of complex Au1)

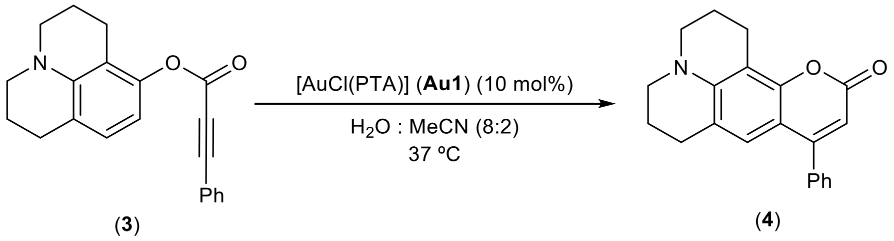

Substrate **3** (0.050 mmol, 15.90 mg) was added to a Schlenk tube containing a stir bar, followed by the addition of [AuCl(PTA)] (**Au1**, 0.005 mmol, 1.90 mg, 10.0 mol%). MeCN (200 µL) was added, and the reaction mixture was stirred at 300 rpm until a homogenous solution was formed (10 s). H_2O (800 µL) was added (final volume 1.0 mL, [Substrate] = 50.0 mM), the Thermowatch-controlled heating block was fixed at 37 °C and the reaction was stirred for 3 h. After this time, the reaction mixture was extracted with CH_2Cl_2 (3 x 10.0 mL) and the combined organic fractions were dried, concentrated over silica and purified by silica flash column chromatography using hexane / EtOAc (8:2) as eluent to obtain the product **4** (0.041 mmol, 13.17 mg, 83%) as a yellow solid.

Supplementary Table 2. Yields obtained in the hydroarylation reaction using different gold complexes.

Entry	[Au]	Yield (%)[a]
1	**Au1**	99 (83)[b]
2	**Au2**	99
3	**Au3**	99
4	**Au4**	99
5	**Au5**	99
6	**Au6**	0
7	**Au7**	70
8	**Au8**	65

[a] Yields by ^{1}H-NMR (using CH_2Br_2 as internal standard). [b] Isolated yield

Representative general procedure for the catalytic hydroarylation in water in presence of additives (exemplified for the use of glycine)

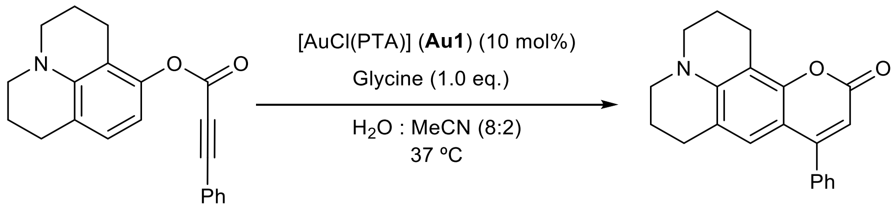

Substrate **3** (0.050 mmol, 15.90 mg) and glycine (0.050 mmol, 3.70 mg, 1.0 eq.) were added to a Schlenk tube containing a stir bar, followed by the addition of [AuCl(PTA)] (**Au1**, 0.005 mmol, 1.90 mg, 10.0 mol%). MeCN (200 µL) was added and the reaction mixture was stirred at 300 rpm until a homogenous solution was formed (10 s). H_2O (800 µL) was added (final volume 1.0 mL, [Substrate] = 50.0 mM), the Thermowatch-controlled heating block was fixed at 37 °C and the reaction was stirred for 24 h. After this time, the reaction mixture was extracted with CH_2Cl_2 (3 x 10.0 mL) and the combined organic fractions were dried to afford the corresponding product **4**.

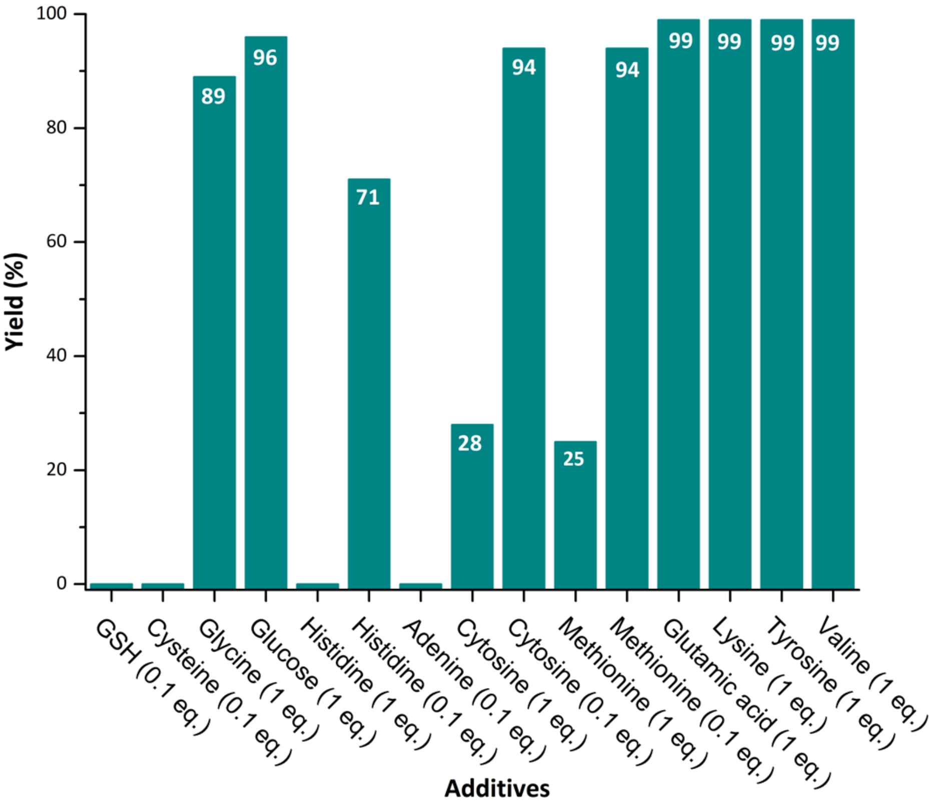

Supplementary Figure 1. Yields by ^{1}H-NMR (using CH_2Br_2 as internal standard) of the hydroarylation reaction in the presence of different additives.

In order to know if the additives that do not affect the reaction were consumed, the crude reaction of the catalytic hydroarylation in the presence of tyrosine was analyzed. After an extraction with CH_2Cl_2 (3 x 10.0 mL) and H_2O (10 mL), both phases were analyzed: the organic layer concentrates were analyzed by ^{1}H-NMR spectroscopy, which confirmed the formation of the expected product **4**. The residue of the aqueous phase was injected (10 µL) in HPLC coupled with an ESI-MS, and the chromatogram obtained (Supplementary Figure 2) essentially shows the expected peak for an intact tyrosine.

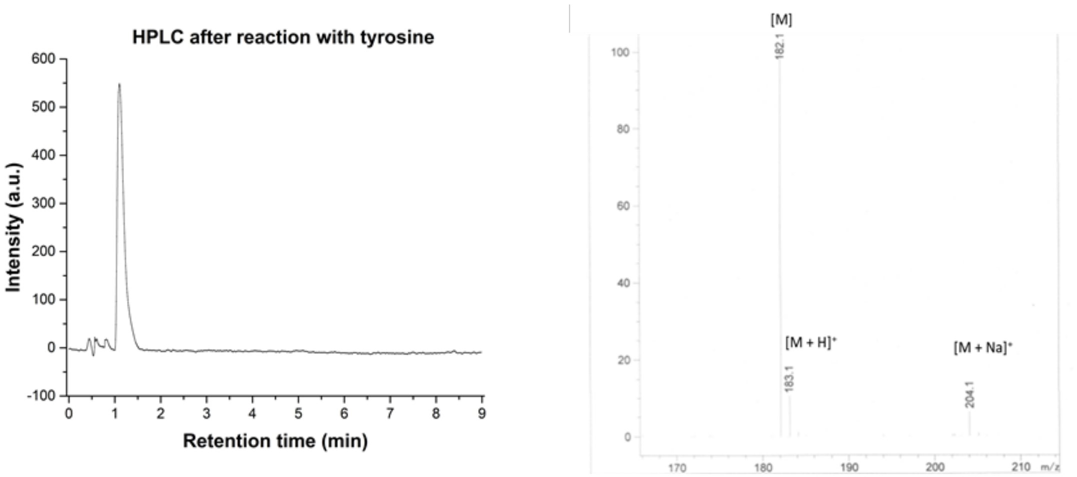

Supplementary Figure 2. Chromatogram and *m/z* of the aqueous phase after the catalytic hydroarylation.

These results confirmed that the additives were not consumed or transformed in other products during the catalytic reaction.

Inhibition by adenine

On the other hand, the observed inhibition by adenine was also investigated by ESI-MS. Thus, in a 1.5 mL HPLC vial the gold complex **Au4** (0.0008 mmol, 0.50 mg) was dissolved in water (1.0 mL) and then adenine (0.008 mmol, 1.1 mg, 10.0 eq.) was added. After 15 min, the mixture was injected (5 µL) in *Bruker Amazon IT/MS*.

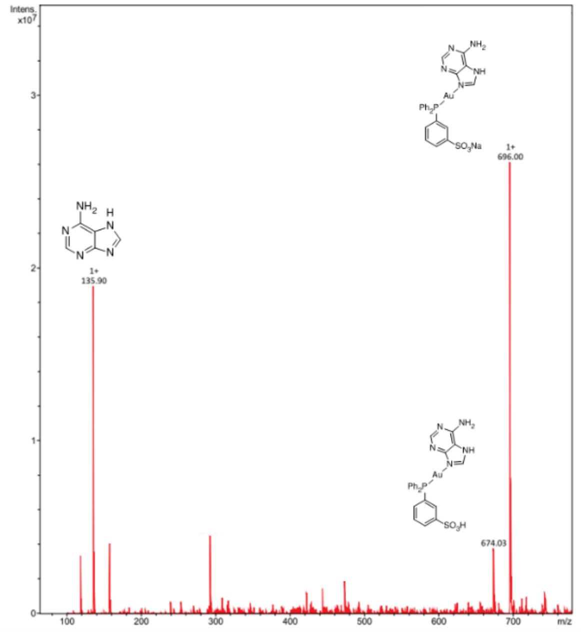

Supplementary Figure 3. ESI-MS spectra of the reaction between the complex **Au4** and adenine.

As can be seen in Supplementary Figure 3, the ESI-MS data confirm that adenine coordinates the gold(I) cation which results from the ionization of **Au4** in water (L-Au^+ species). Thus, two different gold(I)-adenine complexes, depending on whether the sulfinic sodium salt has been protonated (as sulfinic acid) or not, are observed [*m/z* = 674 and 696].

Representative general procedure for the catalytic hydroarylation using different biological media (exemplified for the use of PBS)

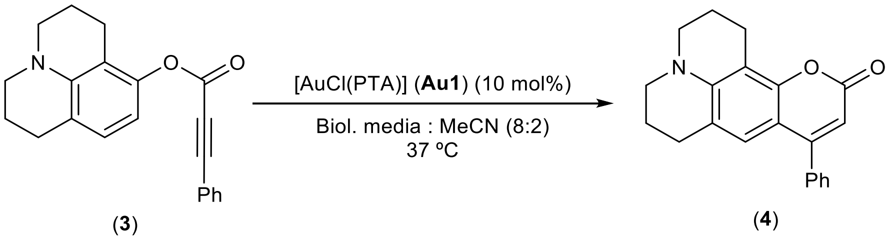

Substrate **4** (0.050 mmol, 15.90 mg) was added to a Schlenk tube containing a stir bar, followed by the addition of [AuCl(PTA)] (**Au1**, 0.005 mmol, 1.90 mg, 10.0 mol%). MeCN (200 µL) was added and the reaction mixture was stirred at 300 rpm until a homogenous solution was formed (10 s). PBS (800 µL) was added (final volume 1.0 mL, [Substrate] = 50 mM), the Thermowatch-controlled heating block was fixed at 37 °C and the reaction was stirred for 24 h. After this time, the reaction mixture was extracted with CH_2Cl_2 (3 x 10.0 mL) and the combined organic fractions were dried to afford the corresponding product **4**.

Supplementary Table 3. Yields of the hydroarylation reaction using different biological media.

Entry	Biological media	Yield (%)[a]
1	PBS	96
2	DMEM	94
3	Lysates[b]	27
4	BSA[c]	93
5	Bacteria	12
6[d]	DMEM	0

[a] Yields by ^{1}H-NMR (using CH_2Br_2 as internal standard). [b] Lysates: 8 mg/mL. [c] BSA: 5 mg/mL. [d] **Au1´** [AuNTf$_2$(PTA)] was used as complex instead of **Au1.**

Catalytic hydroarylation in the presence of DMSO

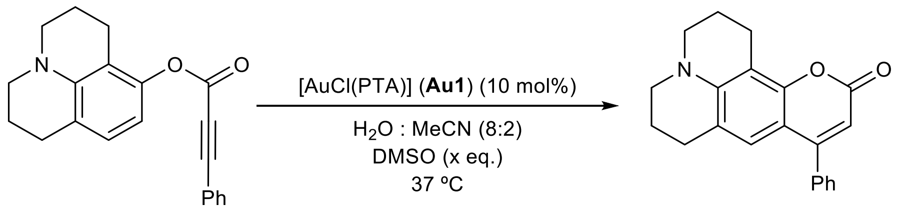

(**3**) (**4**)

Substrate **4** (0.050 mmol, 15.90 mg) was added to a Schlenk tube containing a stir bar, followed by the addition of [AuCl(PTA)] (**Au1**, 0.005 mmol, 1.90 mg, 10.0 mol%). MeCN (200 µL) was added, and the reaction mixture was stirred at 300 rpm until a homogenous solution was formed (10 s). PBS (800 µL) and DMSO (number of equivalents respect to the gold indicated in Supplementary Table 4) were added (final volume 1.0 mL, [Substrate] = 50 mM), the Thermowatch-controlled heating block was fixed at 37 °C and the reaction was stirred for 24 h. After this time, the reaction mixture was extracted with CH_2Cl_2 (3 x 10.0 mL) and the combined organic fractions were dried to afford the corresponding product **4**.

Supplementary Table 4. Yields of the hydroarylation reaction using different amounts of DMSO.

**Entry**	**X eq.**	**µL**	**Yield (%)[a]**
1	10	3.5	99
2	50	17.7	99
3	100	35.5	99

[a] Yields by ^{1}H-NMR (using CH_2Br_2 as internal standard).

SUPPLEMENTARY NOTE 6. Characterization of the new compounds.

NMR Spectra of compound 3.

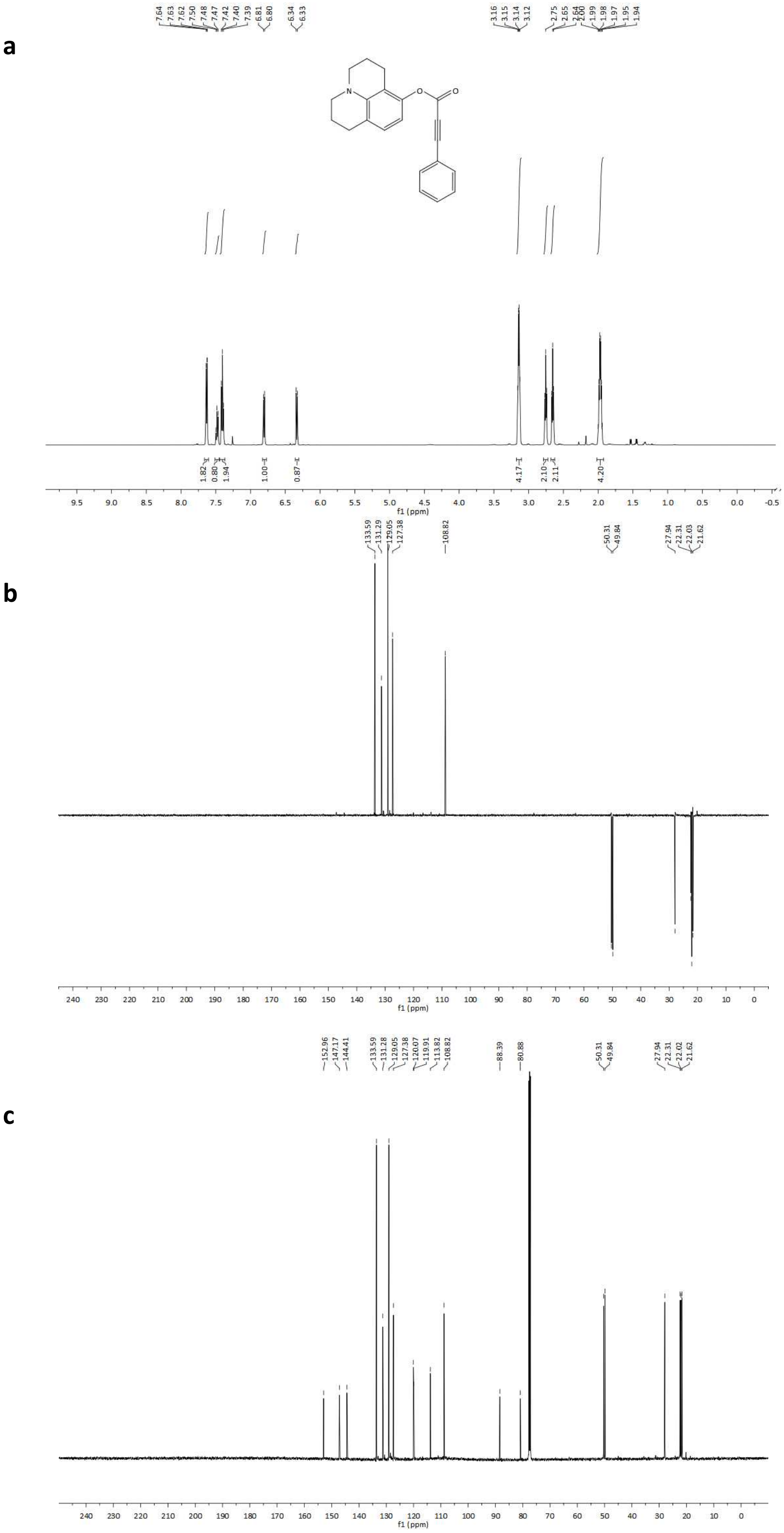

Supplementary Figure 4. NMR spectra of compound **3**. **a,** ^{1}H; **b,** ^{13}C and **c,** DEPT-135.

NMR Spectra of compound 4.

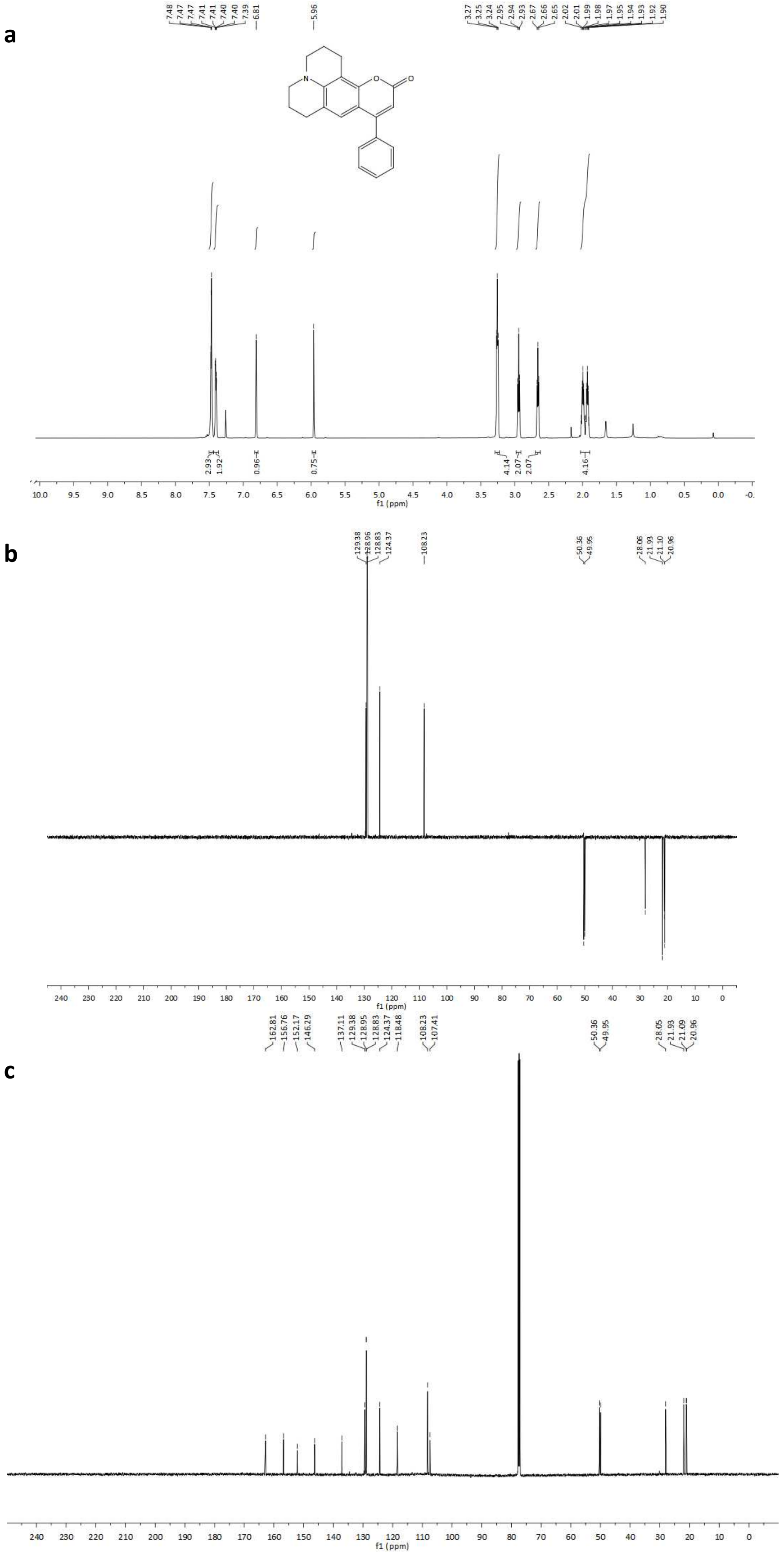

Supplementary Figure 5. NMR spectra of compound **4**. **a,** ^{1}H; **b,** ^{13}C and **c,** DEPT-135.

NMR Spectra of complex Au1´

a

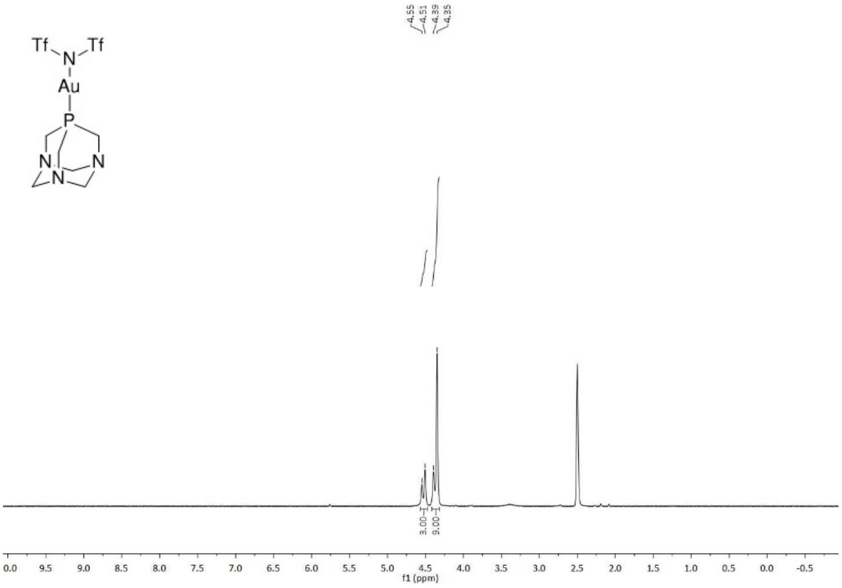

b

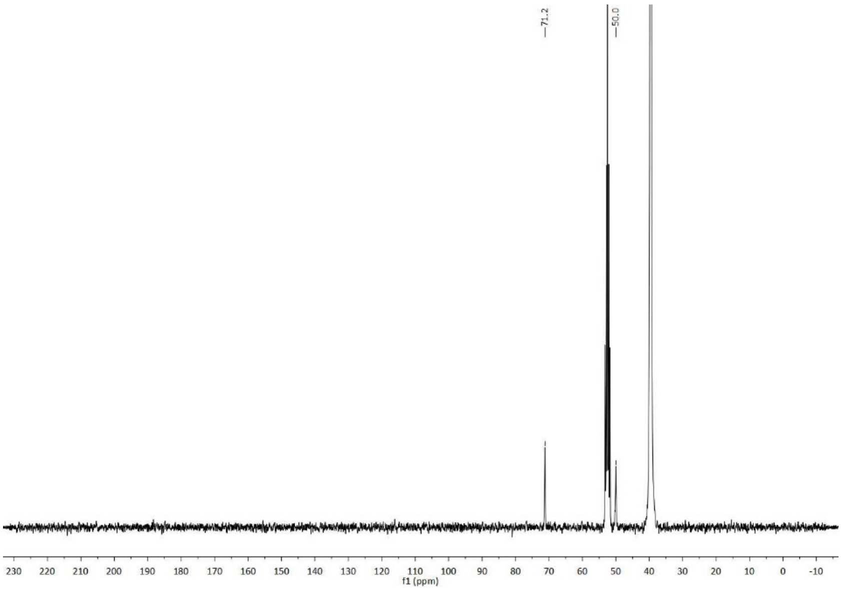

c

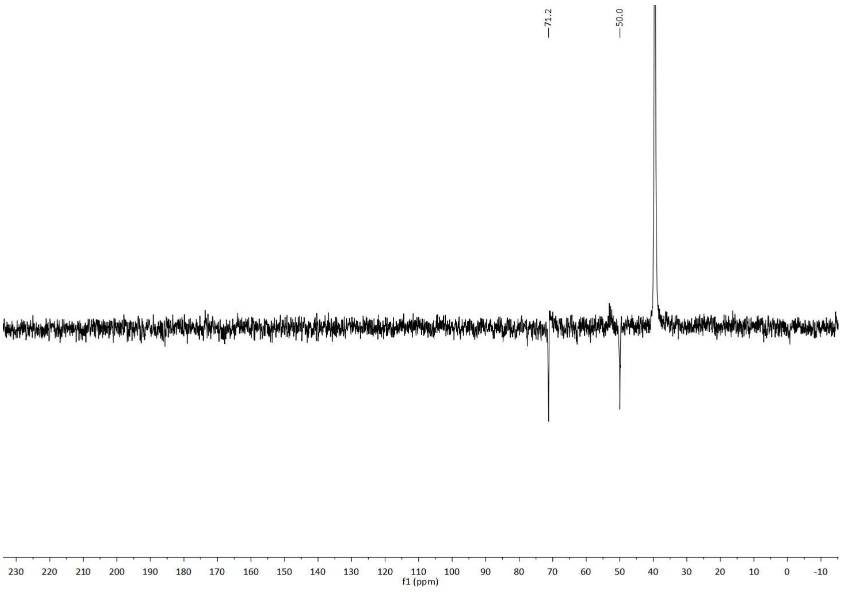

d

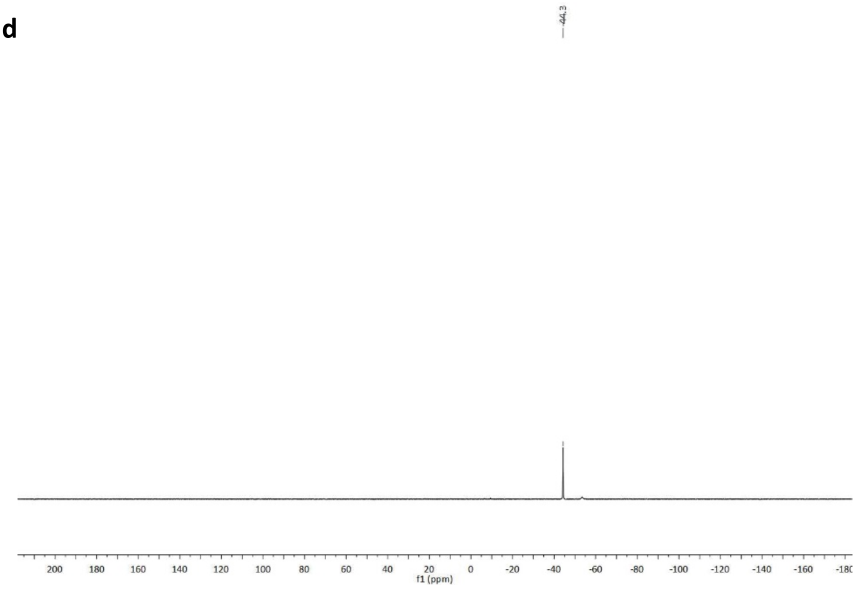

e

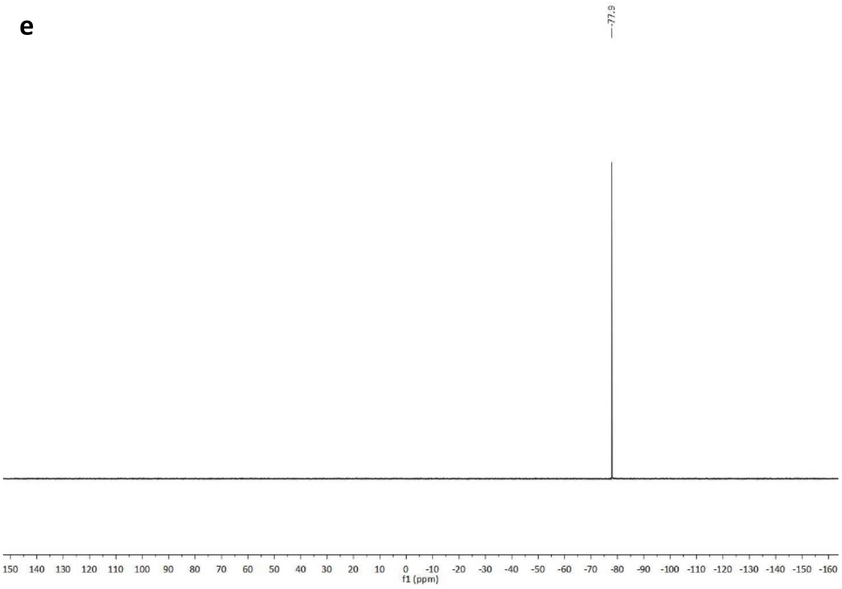

Supplementary Figure 6. NMR spectra of complex **Au1´**. **a,** ^{1}H; **b,** ^{13}C; **c,** DEPT-135; **d,** ^{31}P and **e,** ^{19}F.

NMR Spectra of complex Au4´.

a

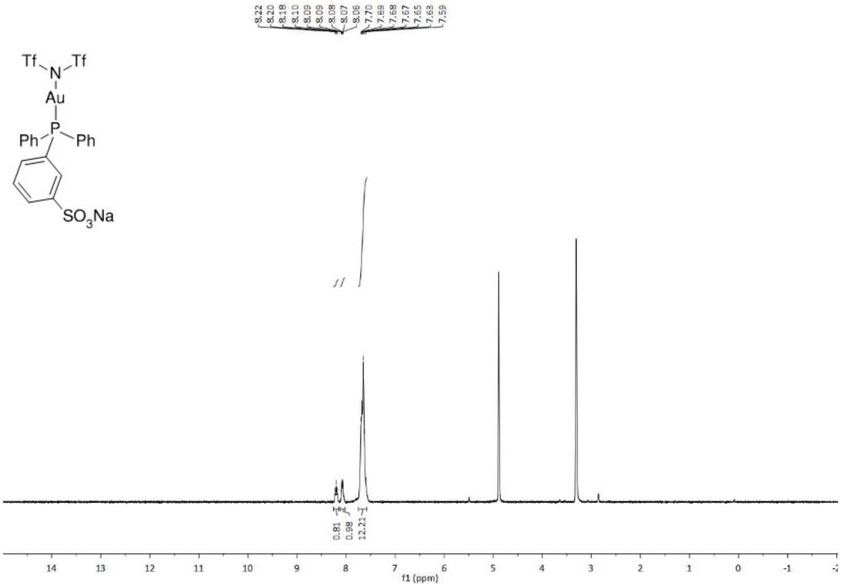

b

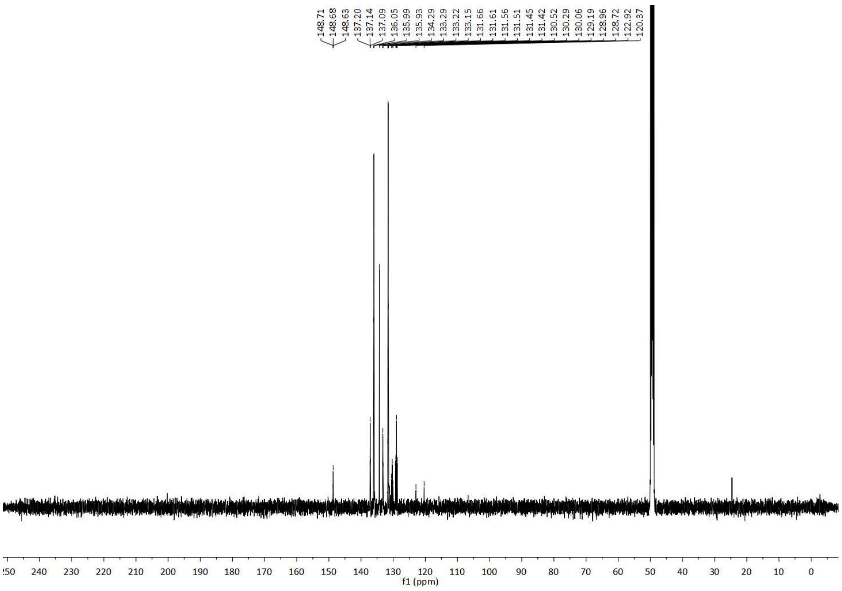

c

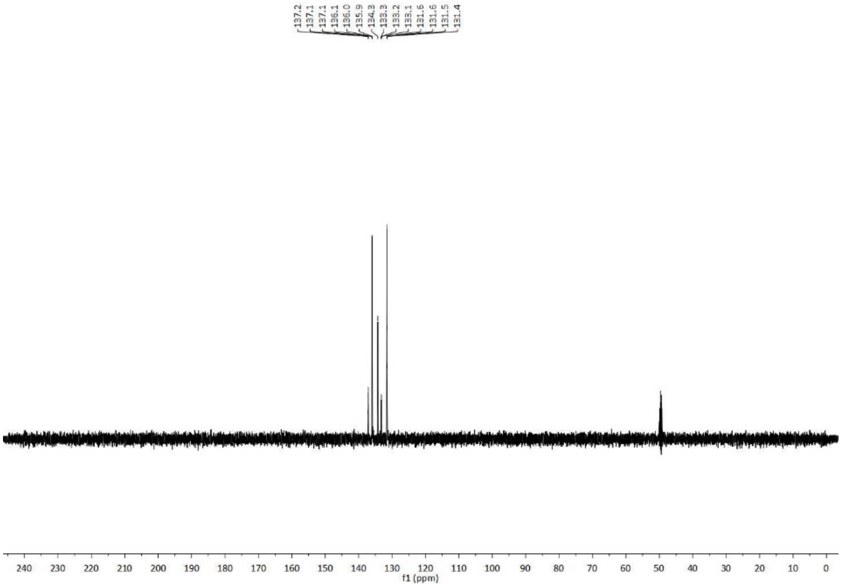

d

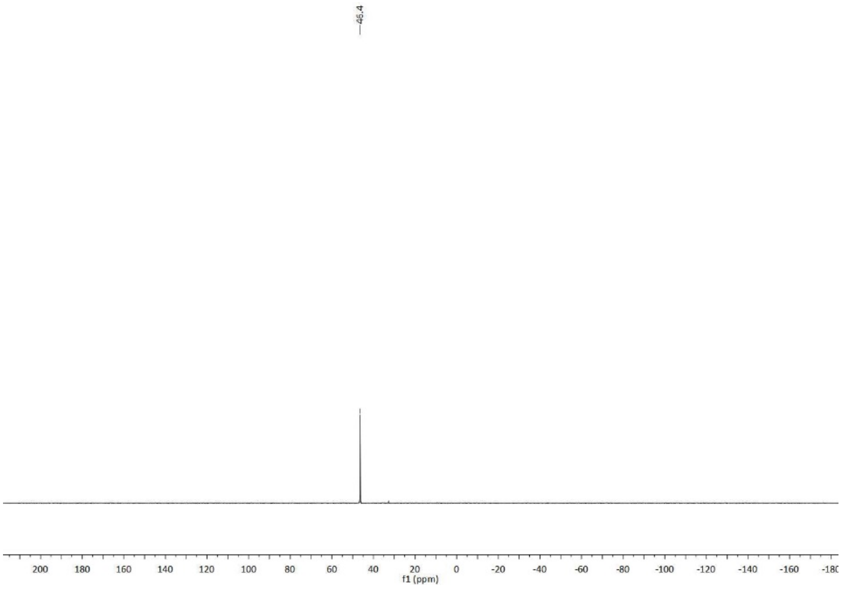

e

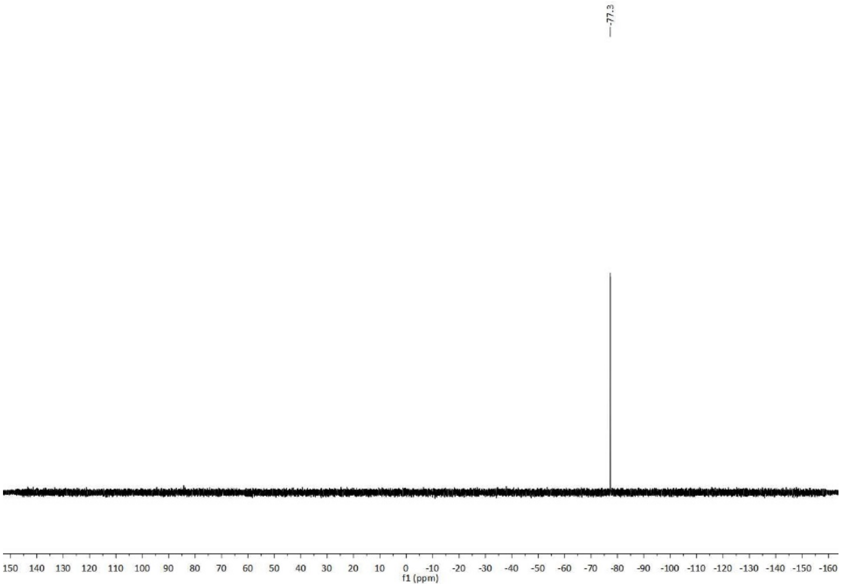

Supplementary Figure 7. NMR spectra of complex **Au4´**. **a,** ^{1}H; **b,** ^{13}C; **c,** DEPT-135; **d,** ^{31}P and **e,** ^{19}F.

UV and fluorescence of compounds 3 and 4

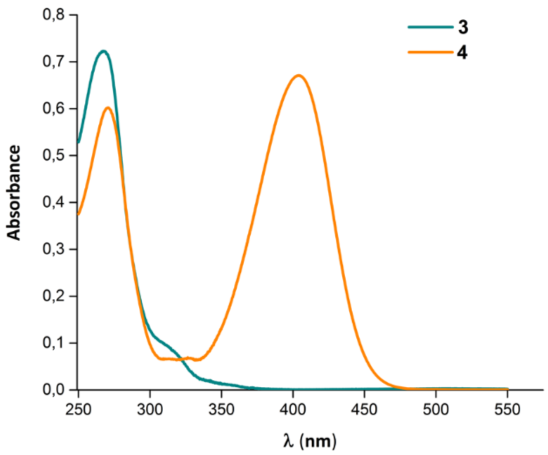

Supplementary Figure 8. Comparison of UV/Vis absorption spectra of **3** (30 µM, grey, λ_{abs} = 266 nm) and **4** (30 µM, orange, λ_{abs} = 402 nm) in EtOH (1.0 mL).

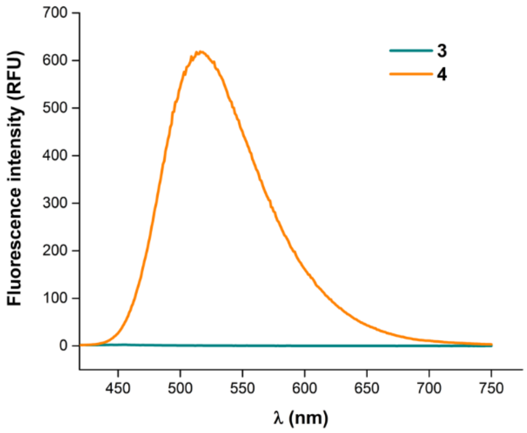

Supplementary Figure 9. Comparison of fluorescence spectra of **3** (5 µM, grey, no emission) and **4** (5 µM, orange, λ_{em} = 515 nm) in EtOH (1.0 mL) with λ_{exc} = 402 nm.

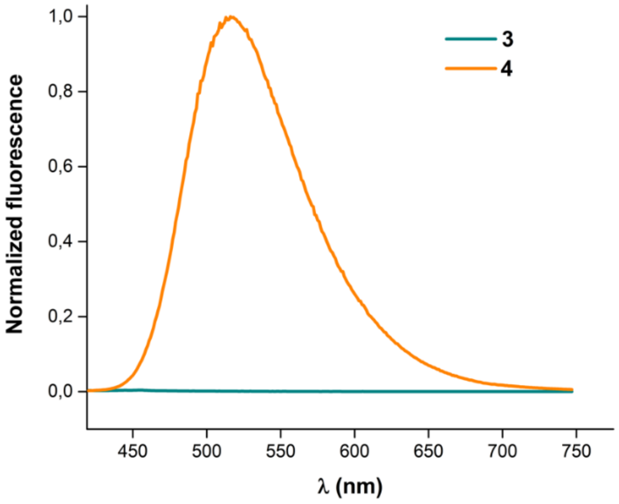

Supplementary Figure 10. Comparison of normalized fluorescence spectra of **3** (5 µM, blue, no emission) and **4** (5 µM, orange, λ_{em} = 515 nm) in EtOH (1.0 mL) with λ_{exc} = 402 nm.

SUPPLEMENTARY NOTE 7. NMR and MS studies on the chloride dissociation in water.

Representative procedure for the ^{31}P-NMR experiments: All the experiments were performed with the complex **Au4** which was added to an NMR tube and dissolved in the corresponding solvent (MeCN or H_2O). Then, the capillary tube with the alendronic acid dissolved in D_2O (used as reference) was added (see Supplemenry Figure 11).

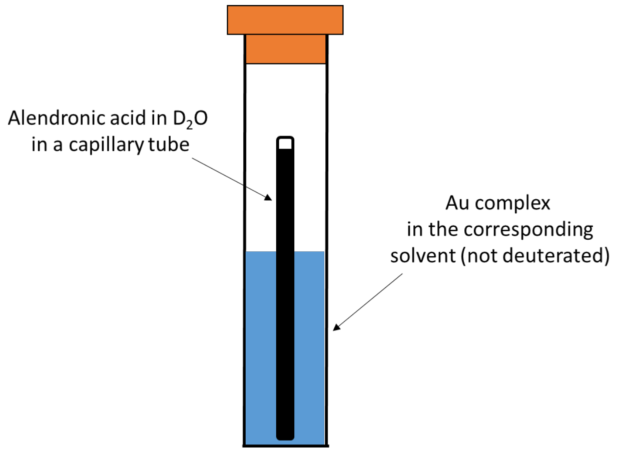

Supplementary Figure 11. Representative procedure for the NMR experiments.

Monitorization of the chloride dissociation

The complex **Au4** was dissolved in 300 µL of H_2O and the ^{31}P-NMR spectra was recorded, observing a signal at 32.96 ppm (Supplementary Figure 12a).

In parallel, we analyzed the samples by ESI-MS: in a 1.5 mL eppendorf the gold complex **Au4** was dissolved in H_2O and the sample (50 µL) was injected in *Bruker Amazon IT/MS*. As it can be observed in Supplementary Figure 12b, the sample in pure water shows four different peaks which correspond to aquo species (*m/z* = 557.0 and 579.0) and chloride complexes (*m/z* = 596.97 and 618.95). Moreover, a sample in pure MeCN was also injected, and only the latter chloride complex was detected (Supplementary Figure 12c).

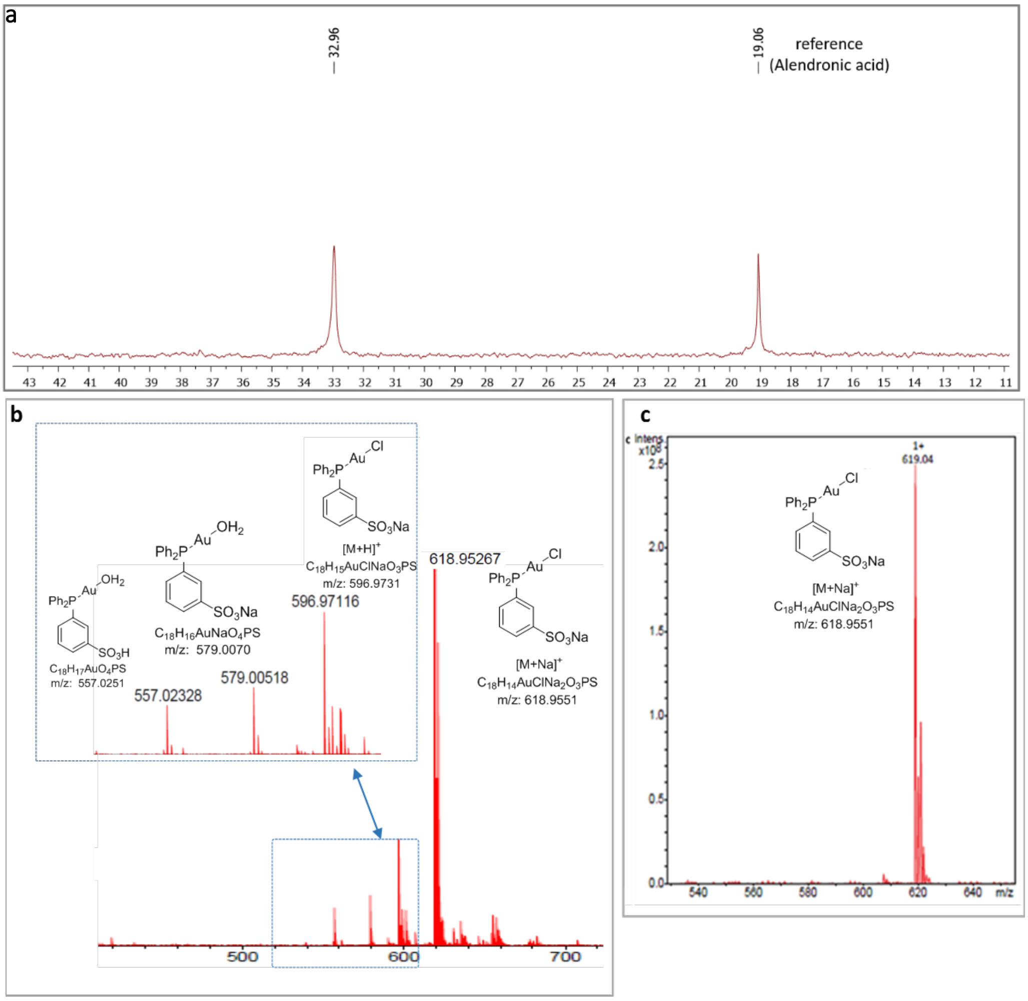

Supplementary Figure 12. ^{31}P-NMR and ESI-MS of the complex Au4. **a**, ^{31}P-NMR spectra of the gold complex **Au4** in H_2O; **b**, ESI-MS spectra of the gold complex **Au4** in H_2O and **c**, ESI-MS spectra of the gold complex **Au4** in MeCN.

<u>Interpretation of Figure 12</u>: These results indicate that gold-aquo species $[Au(OH_2)L]^+$ are generated at some extent when the gold-chloride complex **Au4** is dissolved in water. Accordingly, the ^{31}P-NMR signal at 32.96 ppm might represent the average of these different gold species that are present in equilibrium, in the water solution. On the other hand, when **Au4** is dissolved in MeCN (^{31}P-NMR signal at 35.35 ppm, see Supplementary Figure 15a), the ESI-MS only shows the gold-chloride complex (*m/z* = 618.95, see Supplementary Figure 12c), confirming that in the absence of water the Au-Cl bond is not broken.

Addition of NaCl to the gold complex in water

Increasing amounts of a solution of NaCl (1 M) were added to the NMR tube that contained the solution of **Au4** in water, and the ^{31}P-NMR spectra was recorded. As it is displayed in Supplementary Figure 13a, after sequential addition of 0.5 and 70 equiv. of NaCl, the phosphorus signal shifted from 32.96 ppm to 33.22 and 33.72 ppm, respectively. Finally, when a saturated solution of NaCl was employed as solvent, the signal appeared at 34.06 ppm (all these experiments were performed using alendronic acid as internal standard).

To evaluate whether the change in ionic strength of the solvent (from pure water to saturated NaCl) could be behind of such important shift in the ^{31}P-NMR (from 32.96 to 33.72 ppm), we

measured the ^{31}P-NMR spectra of the corresponding phosphine ligand [3-(diphenylphosphino) benzenesulfonic acid sodium salt] in water and in a saturated solution of NaCl. As it is displayed in Supplementary Figure 14, no changes in the chemical shift were observed, suggesting that the shift is not due to changes in the ionic strength of the media, but instead must arise from the presence of the above mentioned equilibrium of gold-aquo and gold-chloride species.

To further prove this, the addition of NaCl (aq) to **Au4** was also followed by ESI-MS: NaCl was added to a solution of **Au4** in H_2O to reach a final concentration of NaCl of 5 M. After 5 min, the mixture was injected in the *Bruker Amazon IT/MS* and we observed that the signal of the gold(I)-aquo species (*m/z* = 557.0 and 579.0) had essentially disappeared, whereas the peak of the gold-chloride complex (*m/z* = 619.0) substantially increased (Supplementary Figure 13b).

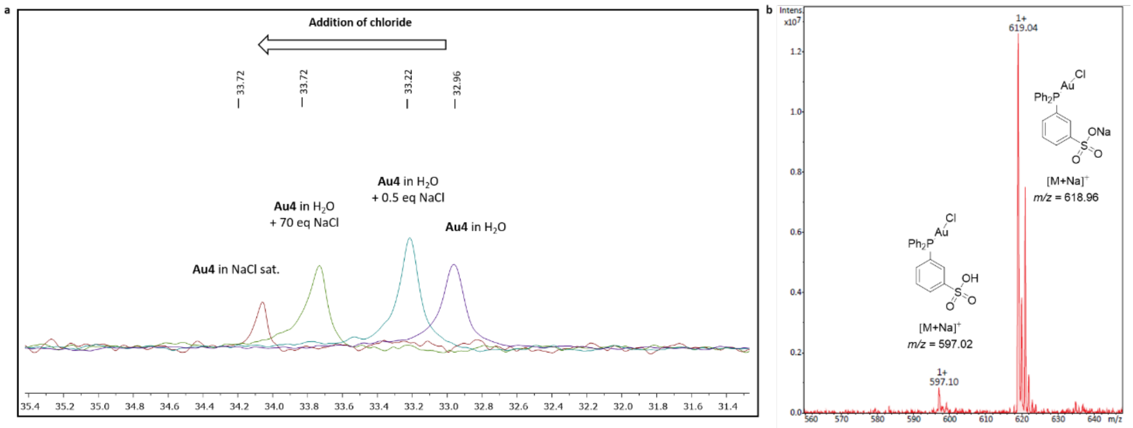

Supplementary Figure 13. ^{31}P-NMR and ESI-MS studies on the addition of NaCl to a solution of complex Au4 in H_2O. **a**, ^{31}P-NMR spectra of the gold complex **Au4** in H_2O, before and after addition of increasing amounts of NaCl and **b**, ESI-MS spectra of the gold complex **Au4** in 5 M NaCl in H_2O.

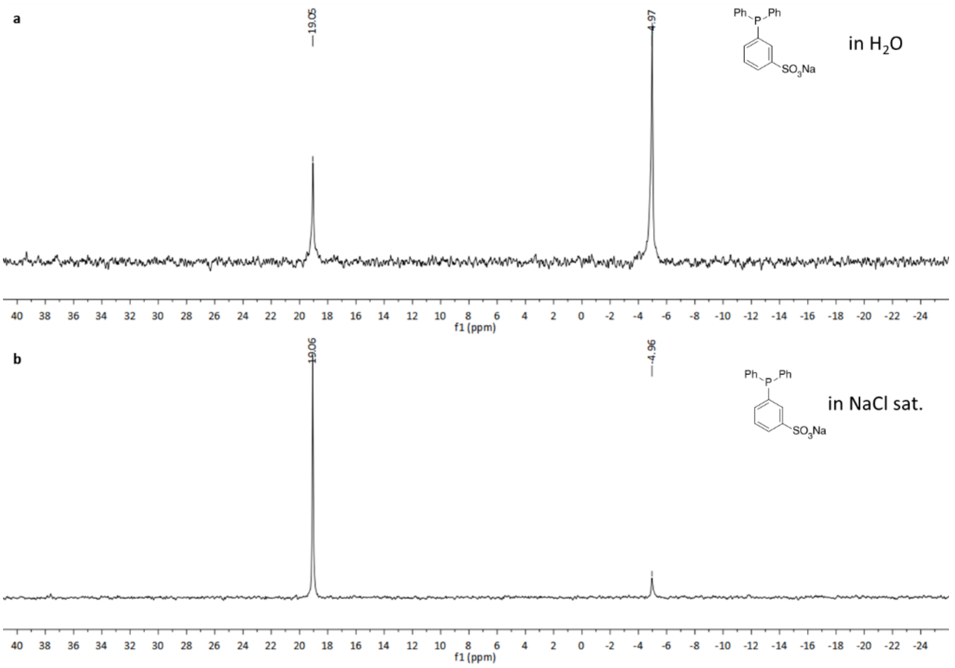

Supplementary Figure 14. ^{31}P-NMR **of** 3-(diphenylphosphino) benzenesulfonic acid sodium salt (the ligand of complex **Au4**) in **a**, H_2O and **b,** in saturated solution of NaCl.

Interpretation of results from Figure 13 and 14: From these results, we deduce that in a saturated solution of chloride anions the proposed equilibrium between the gold(I)-aquo and gold-chloride species of **Au4** is shifted to the latter. Importantly, this hypothesis is consistent with the fact that complex **Au4** is not catalytically active in a 6 M solution of NaCl (see main manuscript), whereas is fully active in water (see Supplementary Table 1, 90% conversion of **1** into **2a** / **2b**) and in water/acetonitrile mixtures (see Supplementary Table 2, 99% conversion of **3** into **4**). In other words, in a saturated solution of NaCl, neutral gold(I) chlorides are predominant.

Monitorization of the hydrolysis of Au4

We have also studied by ^{31}P-NMR and ESI-MS the ionization of **Au4** by adding increasing amounts of water to an acetonitrile solution of **Au4**.

In particular, complex **Au4** was dissolved in MeCN (300 µL) and its ^{31}P-NMR spectra was recorded, observing the expected signal (singlet) at 35.35 ppm (Supplementary Figure 15).

In parallel, we analyzed the samples by ESI-MS: In a 1.5 mL eppendorf the gold complex **Au4** was dissolved in MeCN and a sample (50 µL) was injected in *Bruker Amazon IT/MS*. As it can be observed in Supplementary Figure. 15b, the sample in pure MeCN shows one peak which corresponds to the chloride species (*m/z* = 619.0).

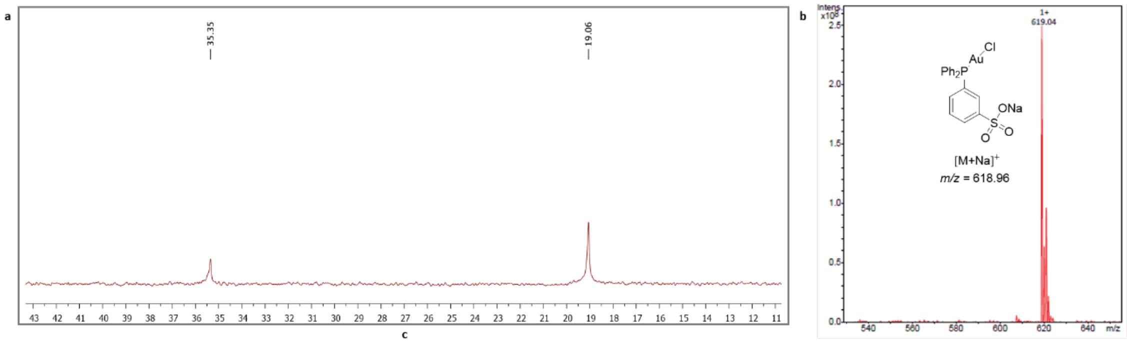

Supplementary Figure 15. ^{31}P-NMR and ESI-MS of complex **Au4** in MeCN. **a**, ^{31}P-NMR spectra of the gold complex **Au4** in MeCN (alendronic acid as internal standard, 19.06 ppm) and **b**, ESI-MS spectra of the gold complex **Au4** in MeCN.

Increasing amounts of H_2O were added to the NMR tube which contained **Au4** in MeCN (50, 100, 200 and 300 µL of H_2O; respectively corresponding to MeCN:H_2O ratios of 7:1, 4:1, 2.5:1 and 1:1). As it is displayed in Supplementary Figure 16, the recorded ^{31}P-signals were 35.26, 35.13, 34.96, 34.84 ppm, respectively. A sample of the complex in a mixture 1:4 MeCN:H_2O (100 µL of MeCN and 400 µL of H_2O) gave a peak of 34.16 ppm. Finally, when the complex was dissolved in H_2O, the ^{31}P-NMR signal appeared at 32.96 ppm.

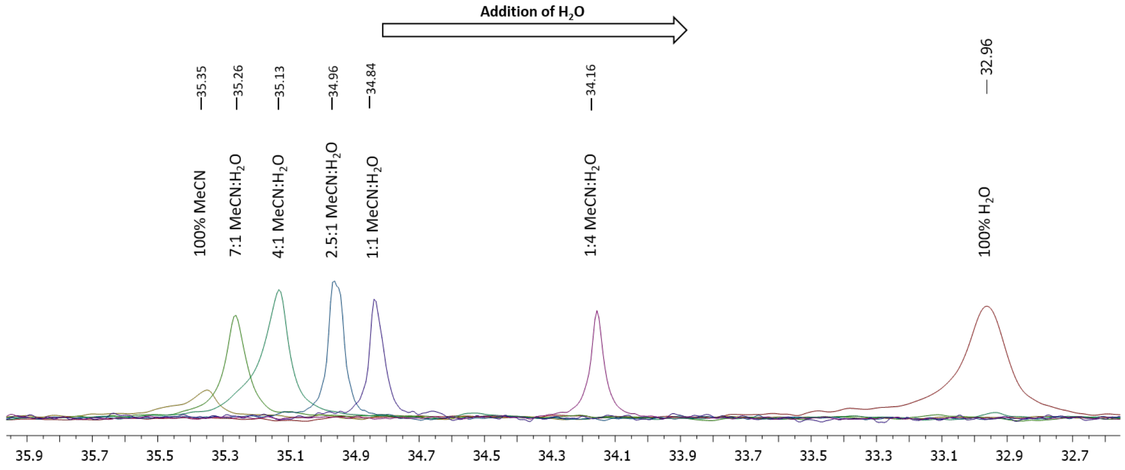

Supplementary Figure 16. ^{31}P-NMR spectra of the gold complex **Au4** in MeCN and after addition of increasing amounts of H_2O (all these experiments were performed using alendronic acid as internal standard).

In parallel, we also monitored the addition of water to an acetonitrile solution of **Au4** by ESI-MS, to identify the species present in the resulting MeCN/H_2O mixtures. Thus, **Au4** was dissolved in 300 µL of MeCN and the sample was injected in the *Bruker Amazon IT/MS*. We observed exclusively the peak of the gold(I)-chloride complex (Supplementary Figure 17). Then, increasing amounts of H_2O were added, and the samples were analyzed by ESI-MS. As can be seen in Supplementary Figure 17, the gold(I)-aquo complex [M^+] can already be detected with a MeCN:H_2O ratio of 4:1 [NOTE: When the amount of water increases this peak becomes more abundant]. Moreover, in addition to this peak, a gold(I)-acetonitrile complex was also detected (*m/z* = 602.09). [NOTE: When **Au4** is dissolved in D_2O/CD_3CN, instead of H_2O/CH_3CN, the analog peaks with deuteriums instead of hydrogens are observed, whereas those corresponding to the gold(I)chloride complexes do not vary].

In conclusion, ^{31}P-NMR and ESI-MS data fully confirm the presence of aquo, acetonitrile and chloride complexes, in different proportions. Moreover, we observe that the presence of water is necessary for the dissociation of the chloride: when **Au4** is dissolved in pure MeCN, the ESI-MS only shows the gold chloride complex (*m/z* = 618.96). Only when water is present, we detect the acetonitrile complex. We propose that water is required for the dissociation of chloride and for triggering the reactivity. We cannot discard associative mechanisms in presence of the alkyne, that further facilitate the dissociation of the chloride in water.

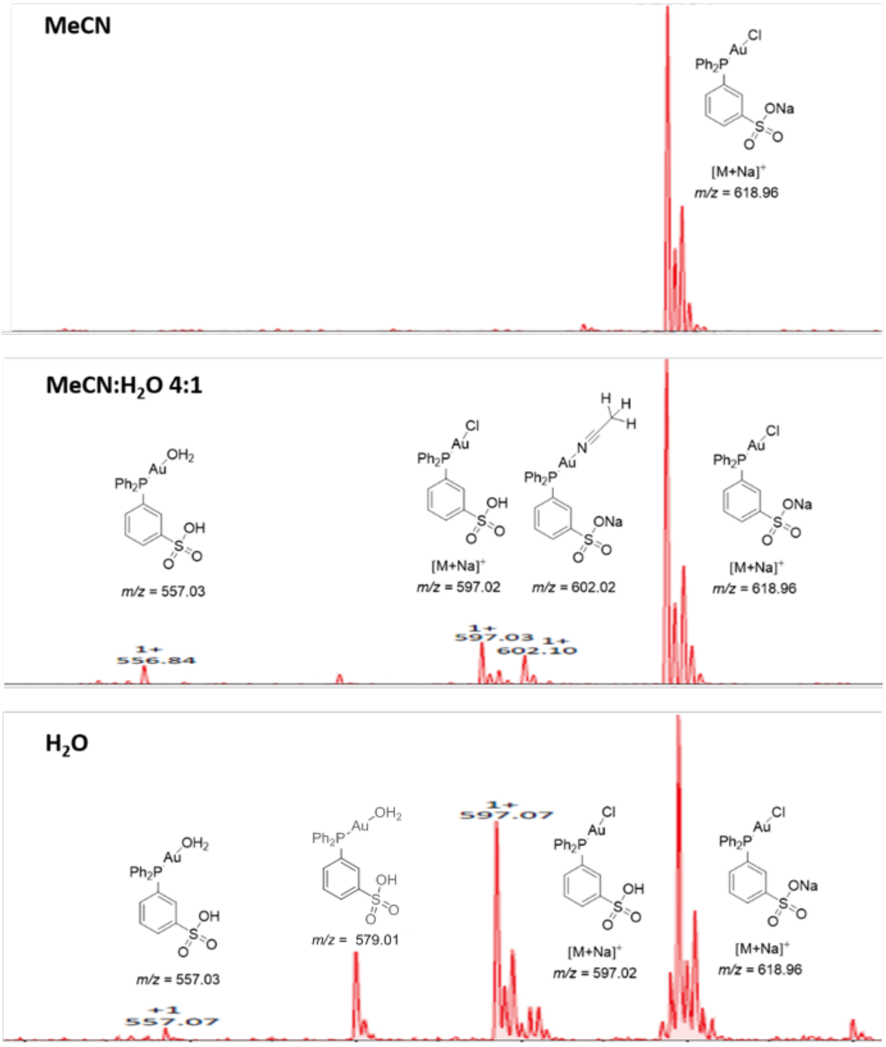

Supplementary Figure 17. ESI-MS spectra of the gold complex **Au4** in MeCN, MeCN:H_2O 4:1 and H_2O.

Coordination of piperidine

Similarly to the studies with acetonitrile, we observed that the gold(I) cationic species generated in situ in presence of water could be trapped by nucleophiles such as piperidine. Control experiments confirmed that these species are not formed using MeCN as solvent.

In two separate 1.5 mL HPLC vials, the gold complex **Au4** (0.0008 mmol, 0.50 mg) was dissolved in dry MeCN (1.0 mL) or water (1.0 mL) and then piperidine (0.017 mmol, 2 µL, 20 eq.) was added to each vial. After 15 min, the mixture (5 µL) was injected in the *Bruker Amazon IT/MS*. As can be seen in Supplementary Figure 18, when water is used as solvent three different cationic gold-piperidine complexes are observed, while the chloride-gold complex remains exclusive in MeCN. This confirms the role of water for the ionization of the Au-Cl bond, which must occur prior to the coordination of piperidine.

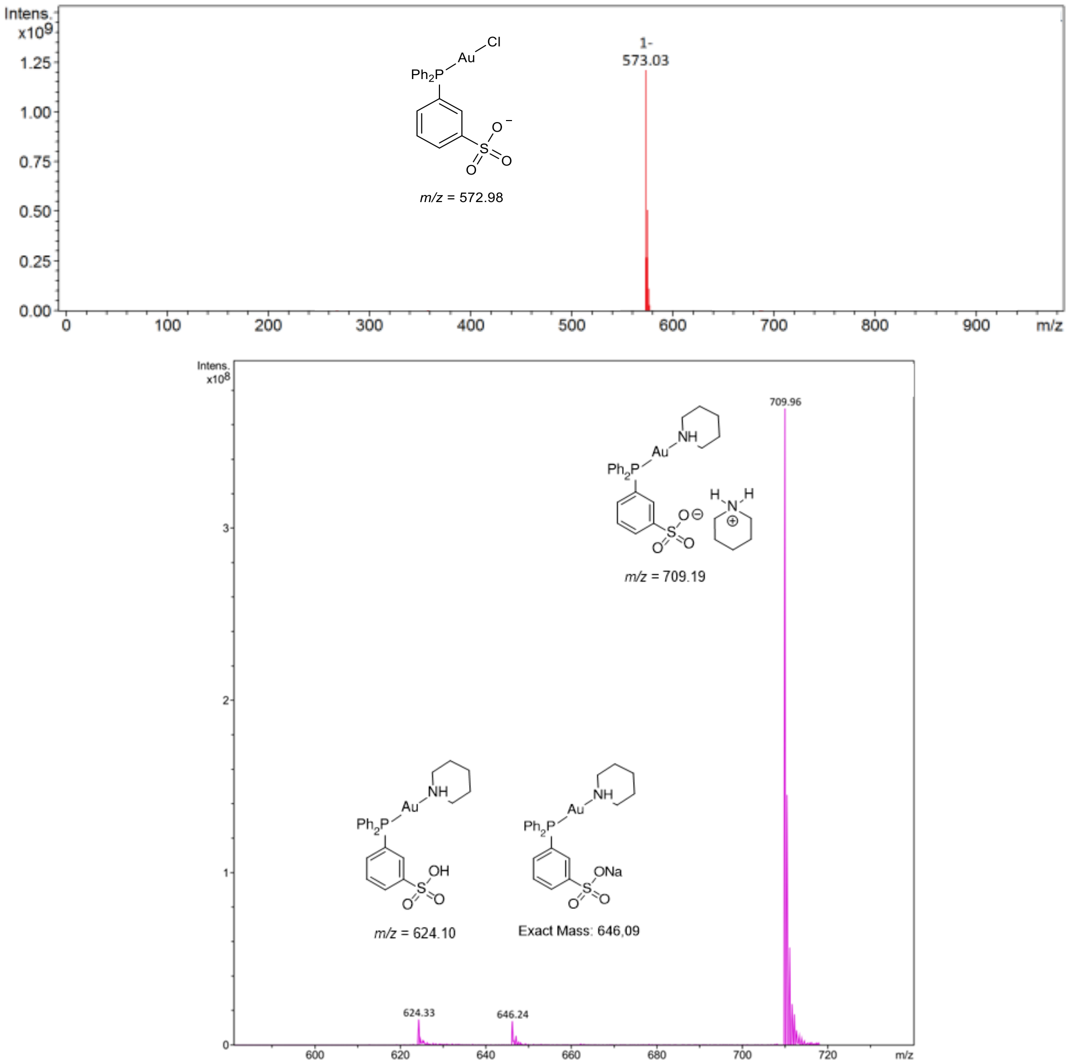

Supplementary Figure 18. ESI-MS spectra of the reaction using gold complex **Au4** and piperidine in MeCN (negative ion MS, top) and H_2O (positive ion MS bottom).

Summary of all the experiments

Thus, complex **Au4** is characterized by a singlet ^{31}P-NMR signal at 35.35 ppm in dry acetonitrile (using alendronic acid as internal standard in a capillary tube with D_2O). Analysis of this acetonitrile solution by ESI-MS reveals a single peak that nicely fits with the molecular structure of **Au4**. However, when the complex is dissolved in water instead of MeCN, the ESI-MS shows peaks corresponding to two main species, the original gold(I)-chloride complex **Au4**, and gold(I)-aquo species ([Au(OH_2)L], L = 3-(diphenylphosphino) benzenesulfonic acid sodium salt). Curiously, the ^{31}P-NMR spectra of a sample of this water solution displays a single peak, a singlet

at 32.96 ppm, suggesting that there is a rapid equilibrium between these gold species.[46] Not surprisingly, when an excess of NaCl is added to the water solution of **Au4**, the resulting ESI-MS and NMR analysis suggest that the equilibrium is shifted to the chloride complex (Supplementary Figure 12a). Furthermore, adding increasing amounts of water to an acetonitrile solution of **Au4** leads to the progressive formation of gold(I)-aquo and gold(I)-acetonitrile species (see Supplementary Figure 16 and 17). Also enlightening, ESI-MS analysis of a mixture obtained after addition of 20 equiv. of dry piperidine to an acetonitrile solution of **Au4** reveals no reaction after 15 min (only **Au4** detected, see Supplementary Figure 18). However, when this experiment is repeated in water, the disappearance of the gold(I)-chloride complex **Au4** and the formation of the corresponding gold(I)-piperidine derivative is observed, a result that is consistent with a water promoted dissociation of the chloride atom, and coordination of piperidine to gold (see Supplementary Figure 18).

All together, these data confirm that water promotes the ionization of the Au-Cl bond and thus drives the complexation of the reactants to the gold(I) complex, which eventually allows to initiate the catalysis.

SUPPLEMENTARY NOTE 8. Studies of the displacement of $[NTf_2]^-$ in Au4´.

The complex **Au4´** was dissolved in dry THF and 5 µL of the solution was injected in the *IT Bruker*. The ESI-MS spectra was recorded. The resulting ESI-MS spectra shows several peaks of multimeric species and fragmentations, that are probably a consequence of the easy dissociation of NTf_2 ligand under the ESI-MS conditions, and formation of different species. However, when 0.5 mL of an aqueous solution of NaCl (5 M) were added to the **Au4´** solution and 5 µL was injected in *IT Bruker*, we could detect the peak corresponding to the expected chloride, which is more stable under the ESI-MS conditions.

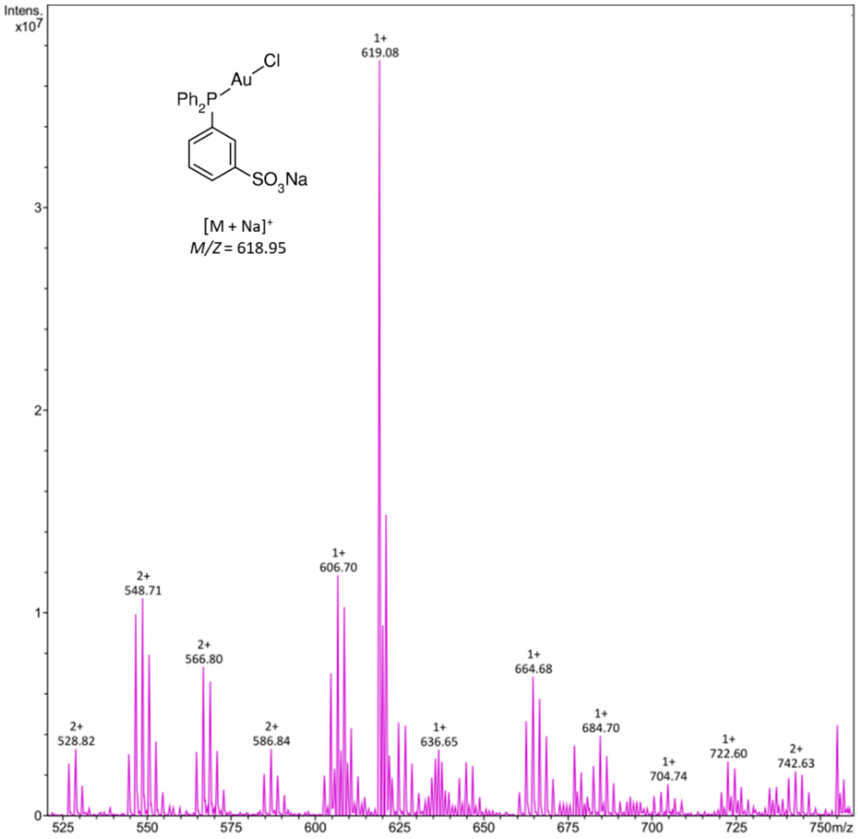

Supplementary Figure 19. ESI-MS spectra of gold complex **Au4´** after addition of NaCl (5 M).

From these results, we deduce that in the presence of NaCl, the $[NTf_2]^-$ is at least partially replaced by the Cl^-.

SUPPLEMENTARY NOTE 9. NMR studies on the solubility of Au6 complex.

An equimolar mixture of **Au1** and **Au6** was introduced in an NMR tube and DMSO-d_6 was used as solvent. ^{31}P-NMR spectrum shows an approximately 1:1 mixture of both complexes after integration of the signals (Supplementary Figure 20a). In MeCN-d_3, the proportion decreases to 1:2 **Au1**:**Au6** (Supplementary Figure 20b). In contrast, in the deuterated mixture of solvents employed for the catalytic reaction, we could only detect the signal of **Au1** (Supplementary Figure 20c). This result reflects the insolubility of **Au6** in the reaction media.

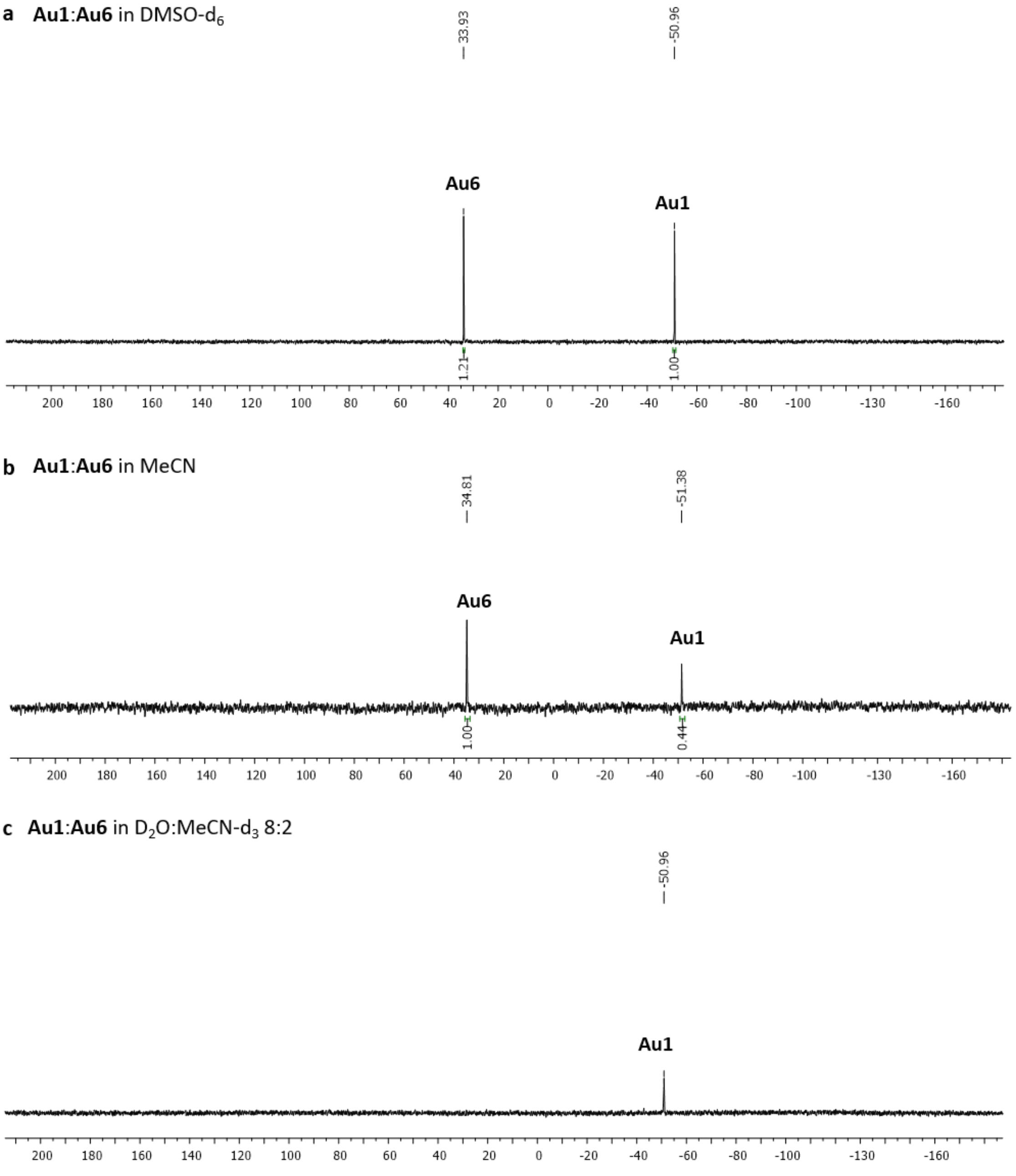

Supplementary Figure 20. ^{31}P-NMR spectra of the gold complexes **Au1** and **Au6** in **a**, DMSO-d_6; **b**, MeCN-d_3; **c**, D_2O:MeCN-d_3 8:2.

These results confirmed that the lack of reactivity of **Au6** in water, and in MeCN/water mixtures (see Supplementary Tables 1 and 2) is due to its lack of solubility, which hinders its water-promoted activation. In fact, in acetonitrile, where **Au6** and the precursor **3** are fully soluble, **Au6** does not catalyzed the conversion of **3** into **4**, unless a chloride scavenger (such as $AgSbF_6$) is used to generate such ionic gold(I) species (e.g. L-Au^+ SbF_6^-).

SUPPLEMENTARY NOTE 10. General information for the biological experiments.

General executions and substances: All steps were performed on a sterile clean bench *Tesltar AV-100* at room temperature. Solutions stored in a fridge were warmed beforehand in a water bath (37 °C). Unless otherwise specified, all incubations were performed in DMEM.

Cell Culture: All cell lines were cultured in DMEM (Dulbecco´s modified Eagle´s medium), 5 mM glutamine, penicillin (100 units mL^{-1}) and streptomycin (100 units mL^{-1}) (all from *Invitrogen*). Proliferating cultures were maintained in a 5% CO_2 humidified incubator at 37 °C.

Fluorescence microscopy: All images were obtained with an *Andor Zyla* mounted on a *Nikon TiE*. Images were further processed with *Image J* or NIS software (Nikon).

Microscopy settings: The parameters of the fluorescent channels were the following:

For the Nikon (Semrock): filter cube DAPI-1160B-000: BP 387/11 nm, LP 447/60 nm and DM 409 nm; filter cube FITC-3540C-000: BP 482/35 nm, LP 536/40 nm and DM 506 nm; filter cube TRITC-B-000: BP 543/22 nm, LP 593/40 nm and DM 562 nm.

SUPPLEMENTARY NOTE 11. Viability assays.

The toxicity of the gold complexes was tested by using of the propidium iodide and MTT assays in HeLa cell line.

Propidium iodide assay:[10] 15000 cells per well were seeded in 96 well plates one day before treatment. Cells were washed once with KRH buffer. To each well, 100 µL of propidium iodide solution in Krebs-Ringer-Hepes buffer (KRH, 50 µM) were then added. After 20 min of incubation at 37 °C, initial fluorescence from each well was measured in a microtiter plate reading spectrophotometer (*Tecan Infinite 200 PRO*, λ_{exc} = 560 nm). Then, different concentrations of the gold complexes were added. Subsequently, fluorescence was measured every 30 min. Between measurements, microtiter plate was incubated at 37 °C. At the end of the experiment, 100 µL of digitonin in KHR (1 mM) was added to each well to permeabilize all cells and label all nuclei with propidium iodide. Fluorescence was measured again to obtain a value corresponding to 100% cell death.

KHR buffer (Krebs-Ringer-Hepes): 136 mM NaCl, 4.7 mM KCl, 1.3 mM $CaCl_2$, 12.5 µM $MgCl_2$, 10 mM Hepes buffer pH = 7.9, 10 mM glucose. Final pH = 7.5.

MTT assay:[11] 15000 cells per well were seeded in 96 well plates 1 day before treatment with different concentrations of the gold complexes (25 µM and 50 µM) in DMEM. After 6 h of incubation, HEPES containing 3-(4,5-dimethylthiazol-2-yl)-2,5-diphenyl tetrazolium bromide (MTT) was added to a final concentration of 0.5 mg mL^{-1}. Cells were then incubated for 4 h to allow the formation of formazan precipitates by metabolically active cells. A detergent solution of 10% SDS and 0.01 M HCl was then added and the plate was incubated overnight at 37 °C to allow the solubilization of the precipitates. The quantity of formazan in each well (directly proportional to the number of viable cells) was measured by recording changes in absorbance at 570 nm in a microtiter plate reading spectrophotometer (*Tecan Infinite 200 PRO*).

a

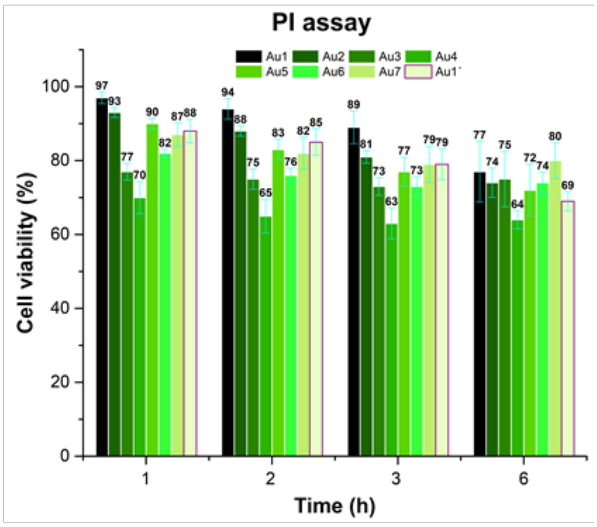

b

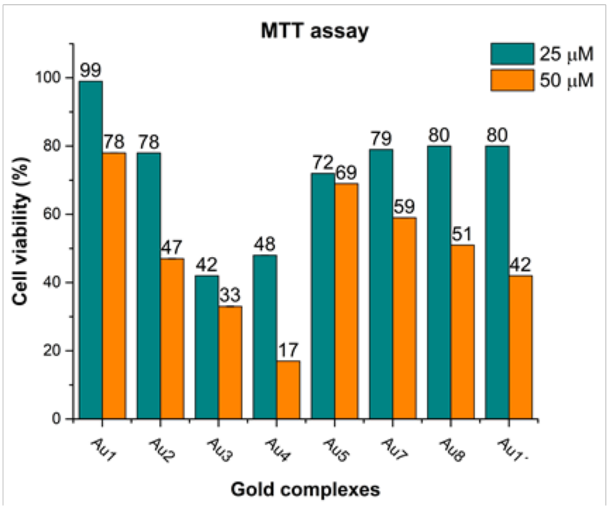

c

Gold complexes and gold salts	IC50 (µM)
Au1	65.2 ± 0.09
Au2	51.7 ± 0.11
Au1’	47.7 ± 0.06
Au3	12.1 ± 0.15
Au4	13.6 ± 0.09
Au5	63.4 ± 0.02
Au7	78.1 ± 0.09
Au8	57.8 ± 0.05

Supplementary Figure 21. Viability Assays. a, Propidium iodide assay. Bars representation of the cell viability with increasing incubation times using 25 µM of the gold complexes. HeLa cells were incubated in KHR buffer containing the indicated amounts of the gold complexes for 6 h. After addition of digitonine, the amount of viable cells was analyzed by fluorescence. **b**, Bars representation of the cell viability with a MTT assay. HeLa cells were incubated in cell culture medium containing the indicated amounts of the gold complexes for 6 h and the amount of viable cells was analyzed by MTT assay. The viability is expressed as the fold change of the fluorescence/absorbance value with respect to untreated cells (value 1.0). Error in these measures was less than 6%. **c**, IC50 values (µM) of the gold complexes and gold salts. The error bars represent the standard deviation of three different samples.

SUPPLEMENTARY NOTE 12. Experiments in living cells.

HeLa cells growing on glass coverslips were incubated with either catalyst **Au1-Au8** (75 µM) for 30 min. Cells were then washed twice with DMEM and incubated with substrate **3** (100 µM) for 6 h. Prior to observation by fluorescence microscopy, the samples were washed twice with fresh DMEM. The coverslips were observed *in vivo* in a fluorescence microscope equipped with adequate filters. Digital pictures of the different samples were taken under identical conditions of gain and exposure.

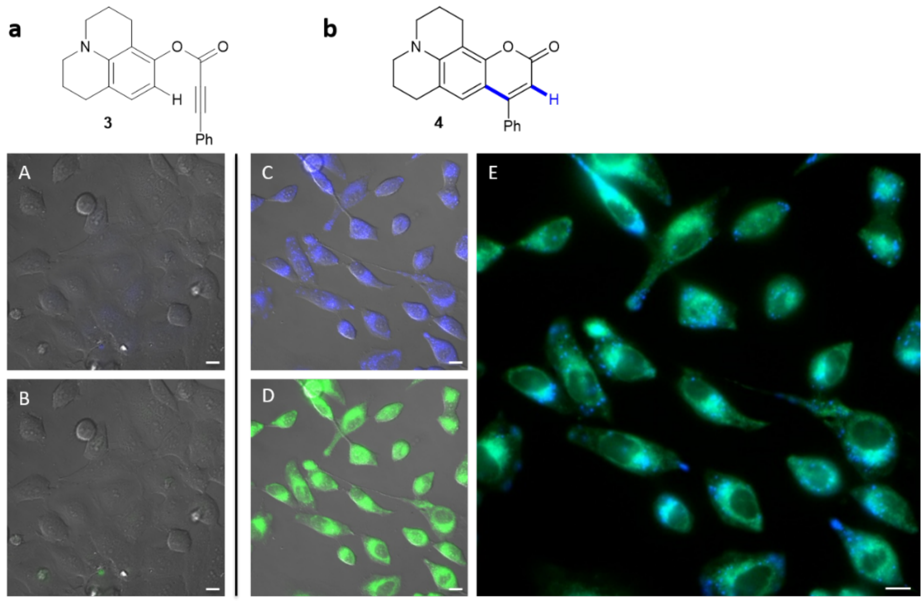

Supplementary Figure 22. Fluorescence and intracellular distribution of substrate 3 and product 4 in HeLa cells. a, Fluorescence micrographies showing the absence of fluorescence in cells treated with precursor **3** and **b**, Fluorescence micrographies showing the fluorescence in cells treated with the product **4**. A) and C) Brightfield and blue channel observation and B) and D) Brightfield and green channel observation; E) Merging of C and B. Reaction conditions: Cells were incubated with either substrate **3** or product **4** (100 µM) for 1 h, followed by double washing with DMEM. Scale bar: 12.5 µm.

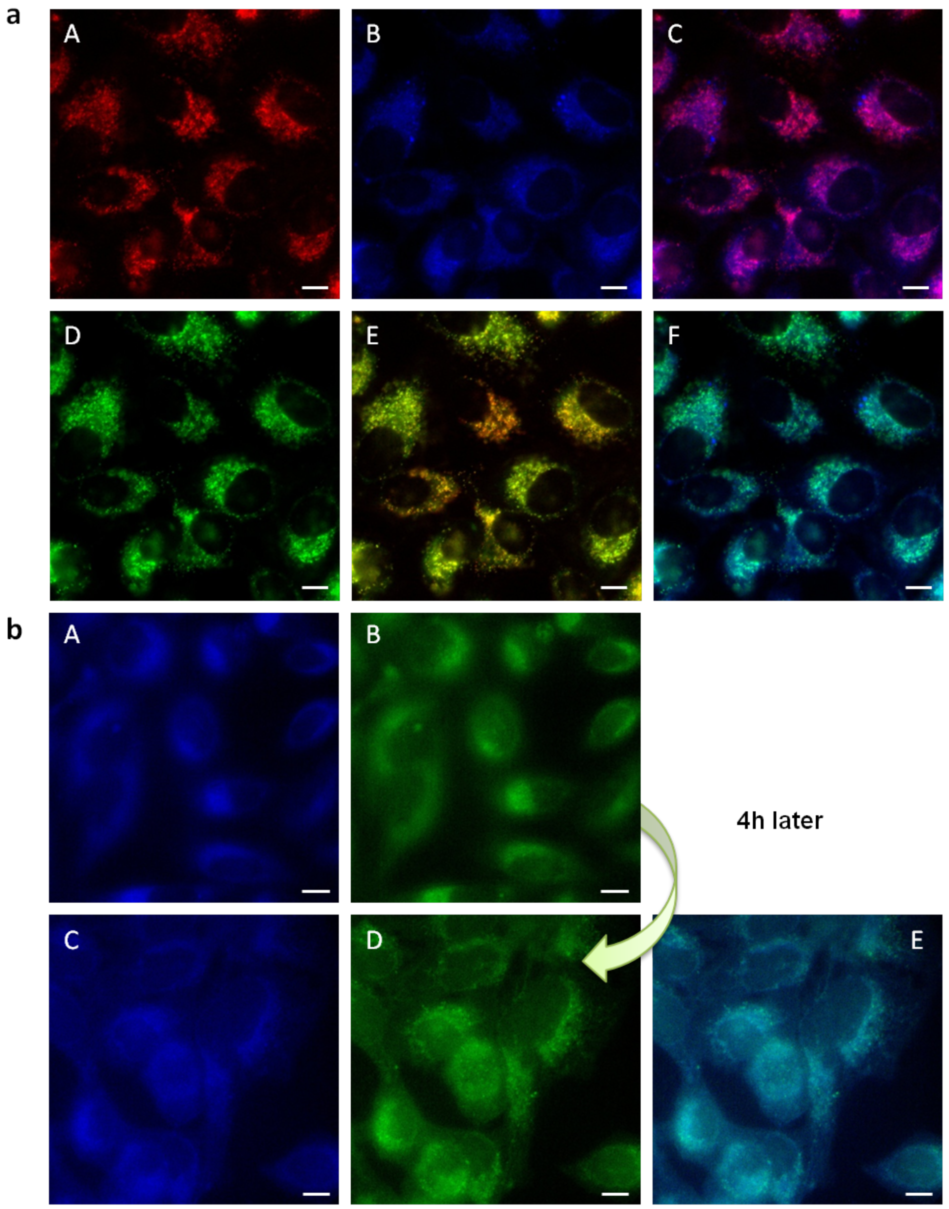

Supplementary Figure 23. Reactivity of gold complex Au2 in HeLa cells. **a**, Catalytic activity of **Au2** and subcellular localization. A) Lysosomal labeling with LysoTracker (red); B) Fluorescence micrographies of cells incubated with the gold catalyst **Au2** followed by addition of substrate **3** (blue); C) Merging of A and B; D) Fluorescence micrographies of cells incubated with the gold catalyst **Au2** followed by addition of substrate **3** (green); E) Merging of A and D; F) Merging of B and D. Reaction conditions: Cells were incubated with the catalyst (50 µM) for 30 min, followed by two washings with DMEM and treatment with substrate **3** (100 µM) for 6 h. Then, cells were incubated with Lysotracker (100 nM) for 15 min and **b**, Changes in fluorescence with increasing reaction time. A, C) Blue fluorescence and B, D) Green fluorescence of cells incubated with the gold catalyst **Au2** followed by addition of substrate **3** (2 h and 6 h, respectively).

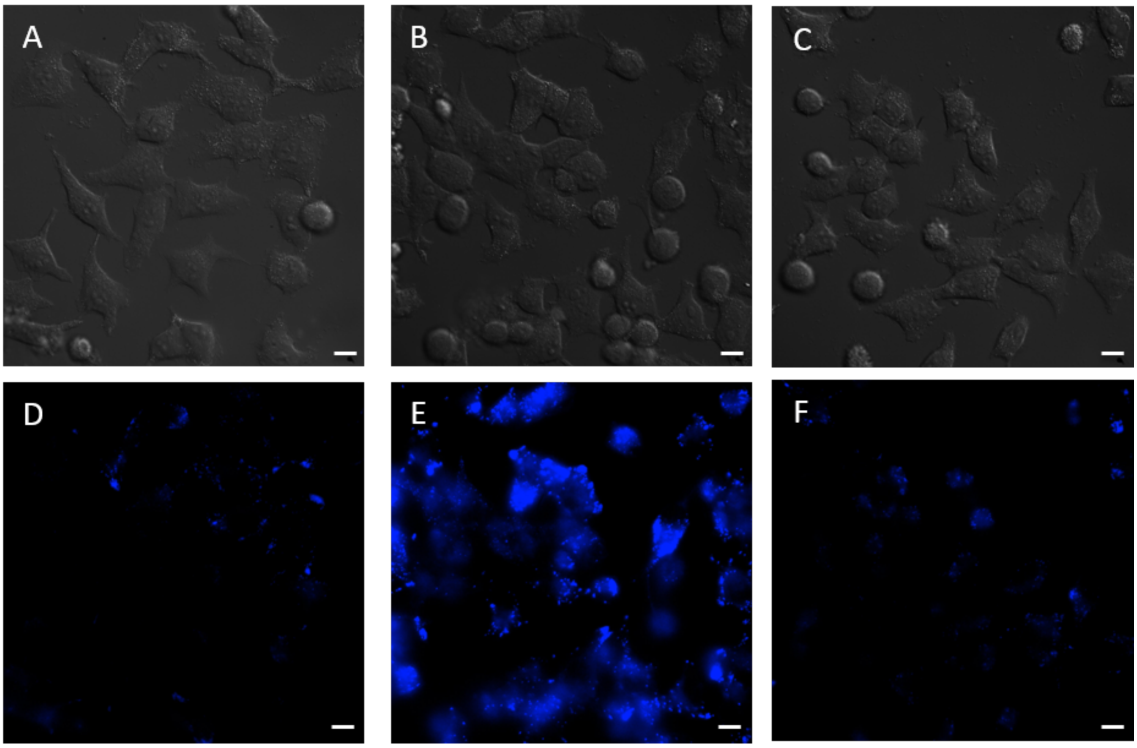

Supplementary Figure 24. Reactivity of gold complex Au1 and Au1’ in HeLa cells (brightfield and blue channel observation). A, D) Fluorescence micrographies of cells incubated with substrate **3**; B, E) Fluorescence micrographies of cells incubated with the gold catalyst **Au1** followed by addition of substrate **3** and C, F) Fluorescence micrographies of cells incubated with the gold catalyst **Au1’** followed by addition of substrate **3**. Reaction conditions: Cells were incubated with the catalyst (50 µM) for 30 min, followed by two washings with DMEM and treatment with substrate **3** (100 µM) for 6 h.

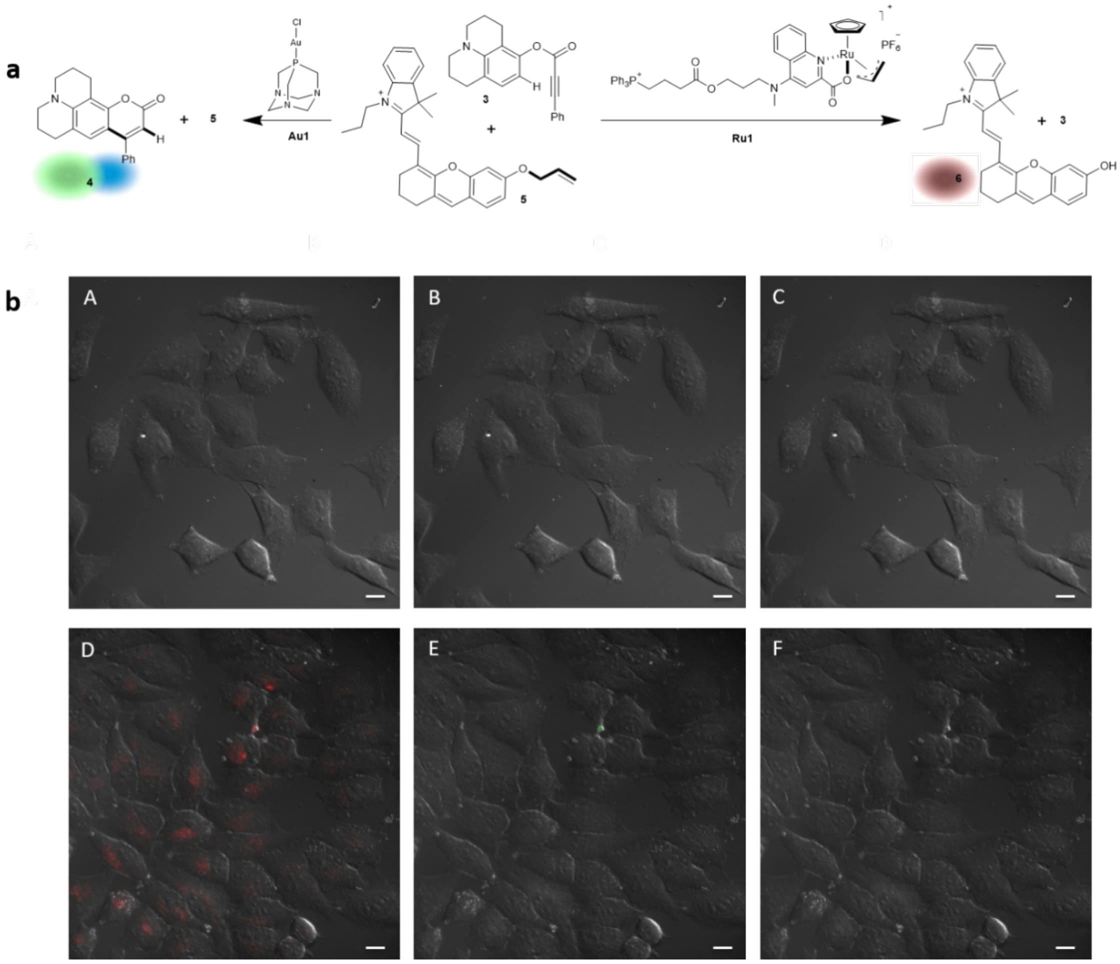

Supplementary Figure 25. Concurrent gold(I) and ruthenium(II) catalysis in HeLa cells. **a**, Schematic representation of the ruthenium(II) and the gold(I) mediated reactions. **b**, Cross control experiments of the catalytic activity of **Ru1** and **Au1** complexes. A, B, C) Red, green and blue fluorescence of cells incubated with **Ru1** followed by addition of substrate **3**; D, E, F) Red, green and blue fluorescence of cells incubated with **Au1** followed by addition of substrate **5**. Reaction conditions: Cells were incubated with **Ru1** (25 µM) or **Au1** (50 µM) for 30 min, followed by two washings with DMEM and treatment with substrate **3** (50 µM) or **5** (100 µM), respectively, for 6 h. Scale bar: 12.5 µm. ICP values: **Ru1** (25 µM, 30 min) = 117.5 ng 10^6 cells^{-1}; Au1 (50 µM, 30 min) = 148.8 ng 10^6 cells^{-1}.

- Calculation of Mander´s coefficients.

The calculation was performed on a dual-color images from fluorescent microscopy experiments. These coefficients were calculated with the public domain tool *JACoP* implemented in the program *ImageJ*.

Mander´s overlap coefficient (MOC) is based on the Pearson´s correlation coefficient but it doesn´t take into account the average intensity values in its mathematical expression.[12] As a result, this parameter is almost independent of signal proportionality and is instead only sensitive to co-ocurrence. MOC varies from 0 to 1, the former corresponding to non-overlapping images and the latter reflecting 100% colocalization between both images. Since MOC is very sensitive to noise, a threshold to the estimated value of background, equalized for every image, was used as zero.

SUPPLEMENTARY NOTE 13. Control reaction between Au1 and substrate 5.

In a Schlenk tube, 100 µL of a solution of substrate **5** (10 mM in MeCN) was added to a 1 mL of H_2O : MeCN (8:2) mixture (final concentration: 1 mM). Then, 100 µL of a solution of complex **Au1** (10 mM in H_2O : MeCN 8:2) was added, the Thermowatch-controlled heating block was fixed at 37 °C and the reaction stirred. The status of the reaction was checked immediately and after 15 h. 100 µL were taken from the solution, diluted with 300 µL of MeCN and filtered through a HPLC-filter in an HPLC vial, and the residue injected in the HPLC *Bruker Amazon IT/MS* using a flow rate of 0.35 mL/min at room temperature using the gradient H_2O/MeCN (95:5) to H_2O/MeCN (5:95) over 14 min.

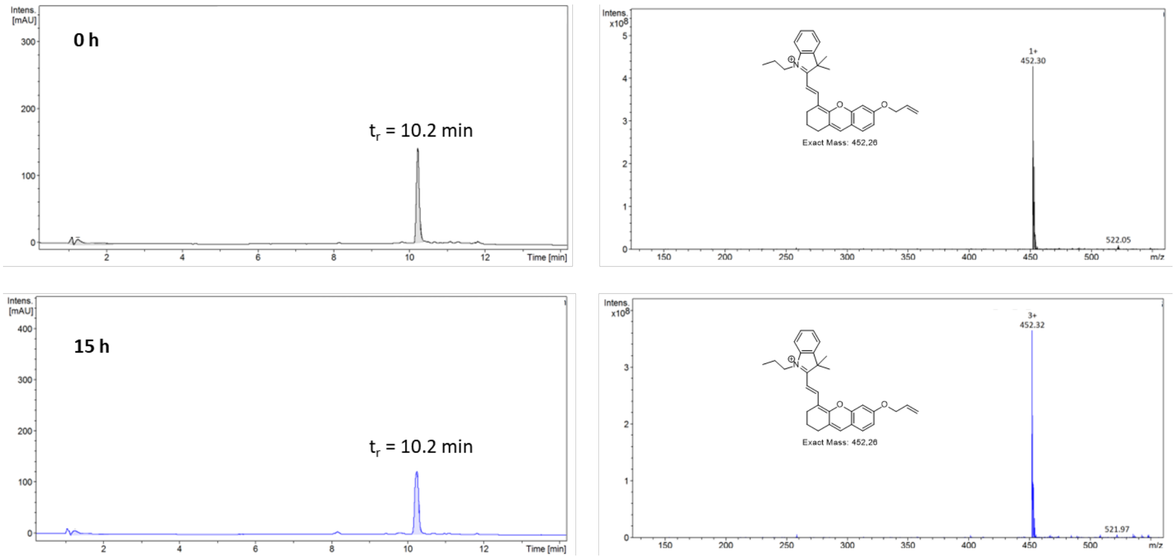

Supplementary Figure 26. Chromatogram (recorded at λ = 330 nm) at 0 h (top) and after 15 h (bottom) of the reaction between substrate **5** with one equivalent of **Au1** and the corresponding *m/z* of the peak at 10.2 min.

The results confirmed that the **Au1** cannot promote the deallylation reaction of the substrate **5**.

SUPPLEMENTARY NOTE 14. ICP analysis

For the ICP measurements, a total of 3 x 10^6 HeLa cells growing in 6 well plates were treated with 75 µM of the different gold complexes in DMEM for 1 h. Prior to digestion, the samples were washed with fresh DMEM and then twice with PBS. The obtained fractions were digested in duplicate in HNO_3/H_2O_2 by microwave heating and analyzed by ICP-MS.

SUPPLEMENTARY NOTE 15. Flow cytometry studies.

After the incubation time, cells were washed twice with PBS, harvested with trypsin/EDTA for 15 min and resuspended in 2% FBS in PBS buffer with 5 mM EDTA. The fluorescence results of the intracellular reactions were analyzed by flow cytometry. As observed in Figure 4, quantification by flow cytometry showed fluorescence corresponding to the product using the Green-B emission filter (512/18 nm).

SUPPLEMENTARY NOTE 16. Estimation on the turnover number in cells.

CAUTION: These “quantitative” results should be considered just indicative, and not be overinterpreted. Working with millions of living cells that do not always have the same confluence or shape, and using experimental protocols that include washing, extraction or cell counting steps, etc. can introduce significant errors.

In addition, the amount of gold considered is that resulting from ICP-MS measurements, which not necessarily correlates with the real amount of active complex inside cells; and we do not know the amount of substrate that is uptaken by the cells.

General procedure: The hydroarylation reaction of substrate **3** was quantified by fluorescence measurements using a *Varian Cary Eclipse* fluorometer.

The experiments were performed in plates of 100 mm as follows:

100000 cells per well were seeded in 100 mm plates two days before treatment with the gold(I) complex and the substrate **3**. For each measurement, four plates were used.

Cells growing on plate of 100 mm were incubated with **Au1** (15 µL, 50 µM) in 3 mL of DMEM for 30 min. Cells were then washed twice with DMEM and incubated with substrate **3** (30 µL, 100 µM) for 6 h. Prior to extraction, the samples were washed with 2 mL of DMEM followed by two washes with PBS (2 mL). Then 1 mL of MeOH in water was added. After 5 min and pipetting up, this solution was transferred to a 1.5 mL eppendorf. Finally, we obtained 5 mL of extracts from the four plates we used.

To normalize the results, the number of cells was measured after the experiments. After the incubation time, cells were washed twice with 2 mL of PBS, harvested with 1 mL of trypsin/EDTA for 5 min at 37 °C and resuspended in 2 mL of DMEM buffer. An aliquot of 50 µL was transferred to a 1.5 mL Eppendorf and diluted with 450 µL of PBS. Then the number of cells was measured with *ScepterTM 2.0 Handheld Automated Cell Counter*.

Calibration: Calibration experiments were performed in a *Varian Cary Eclipse* fluorometer by changing the concentration of the product **4**, varying from 1 µM to 0.4 µM. The experiments were performed by duplicate and the fluorescence measured using the following settings: increment 1.0 nm, averaging time 0.1 s, excitation slit width 5.0 nm, emission slit width 10.0 nm, PMT voltage 680 V (excitation: 402 nm, emission: 512 nm). The fluorescence results were processed with *OriginPro 2016* to obtain the calibration equation, which was applied to quantify the fluorescence of the experiments in living cells.

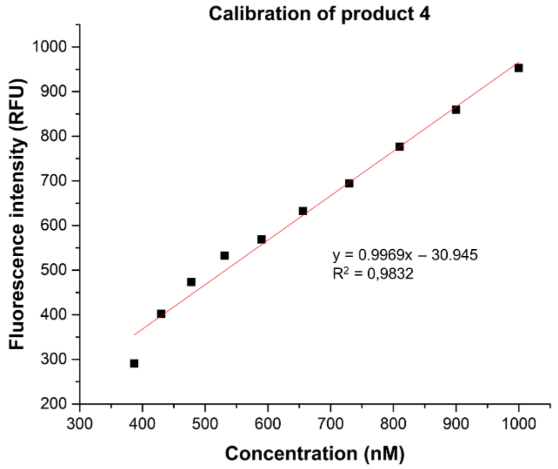

Supplementary Figure 27. Calibration curve of the fluorescent reaction product **4**. Fluorescence intensity (excitation: 402 nm, emission: 512 nm) at different concentrations. Linear regression curve for 357-1000 nm is shown.

Numbers of cells per plate in experiments with **Au1**: 1000000 cells per plate.

ICP value: 195.89 ng of Au 10^6 cells^{-1}. Which means 5.03 x 10^{-11} mol Au 10^6 cells^{-1}.

Fluorescence intensity (using 4 plates): 441.09.

Using this value, the number of mols of product generated is 2.263 x 10^{-10}. Normalizing taking into account the number of cells used (4000000 of cells), the value is 5.66 x 10^{-11} 10^6 cells^{-1}.

Estimated TON = mol product / mol Au = 1.12.

SUPPLEMENTARY REFERENCES.

[1] Kemper, B.; Hristova, Y. R.; Tacke, S.; Stegemann, L.; van Bezouwen, L. S.; Stuart, M. C. A.; Klingauf, J.; Strassert, C. A. & Besenius, P. Facile synthesis of a peptidic Au(I)-metalloamphiphile and its self-assembly into luminescent micelles in water. *Chem. Commun.* **51**, 5253-52556 (2015).

[2] Rodríguez-Álvarez, M. J.; Vidal, C.; Díez, J. & García-Álvarez, J. Introducing Deep eutectic solvents as biorenewable for Au(I)-catalysed cycloisomerization of γ-alkynoic acids: an unprecedented catalytic system. *Chem. Commun.* **50**, 12927-12929 (2014).

[3] Kriechbaum, M.; List, M.; Himmelsbach, M.; Redhammer, G. J. & Monkowius, U. Peptide coupling between amino acids and the carboxylic acid of a functionalized chloride-gold(I) phosphine. *Inorg. Chem.* **53**, 10602-10610 (2014).

[4] Sanz, S.; Jones, L. A.; Mohr, F. & Laguna, M. Homogenous catalysis with gold: efficient hydration of phenylacetylene in aqueous media. *Organometallics* **26**, 952-957 (2007).

[5] Palanichamy, K. & Ontko, A. C. Synthesis, characterization, and aqueous chemistry of cytotoxic Au(III) polypyridyl complexes. *Inorg. Chim. Acta.* **359**, 44-52 (2006).

[6] Mézailles, N.; Ricard, L. & Gagosz, F. Phosphine gold(I) bis-(trifluoromethanesulfonyl)imidate complexes as new highly efficient and air-stable catalysts for the cycloisomerization of enynes. *Org. Lett.,* **7**, 4133-4136 (2005).

[7] Do, J. H.; Kim, H. N.; Yoon, J.; Kim, J. S. & Kim, H.-J. A rationally designed fluorescence turn-on probe for the gold(III) ion. *Org. Lett.* **12**, 932-934. (2010).

[8] Vadola, P. A. & Sames, D. Catalytic coupling of arene C-H bonds and alkynes for the synthesis of coumarins: substrate scope and application to the development of neuroimaging agents. *J. Org. Chem.* **77**, 7804-7814 (2012).

[9] Wu, X.; Li, L.; Shi, W.; Gong, Q. & Ma, H. Near-infrared fluorescent probe with new recognition moiety for specific detection of tyrosinase activity: design, synthesis, and application in living cells and zebrafish. *Angew. Chem. Int. Ed.* **55**, 14728-14732 (2016).

[10] Riccardi, C. & Nicoletti, I. Analysis of apoptosis by propidium iodide staining and flow cytometry. *Nat. Protoc.* **1**, 1458-1461 (2006).

[11] Berridge, M. V.; Herst, P. M. & Tan, A. S. Tetrazolium dyes as tools in cell biology: new insights into their cellular reduction. *Biotechnol. Annu. Rev.* **11**, 127-152 (2005).

[12] Manders, E. M. M.; Verbeek, F. J. & Ate, J. A. Measurement of co-localization of objects in dual-colour confocal images. *J. Micros.* **169**, 375-382 (1993).
